# Supplementary material for: Identification and Treatment of Opioid Withdrawal and Opioid Use Disorder in the Emergency Department
Source: MedEdPORTAL. 2020 May 15;16:10899. doi: 10.15766/mep_2374-8265.10899 (PMC7331957; doi:10.15766/mep_2374-8265.10899)
Supplement: Supplementary file 1 — OUD in the ED Introduction.pptxOUD Case - Facilitator.docxOUD Case - Trainee.docxTest Questions.docxTest Questions Answer Key.docx [file mep_2374-8265.10899-s001.zip › A. OUD in the ED Introduction.pptx]

## Slide 1
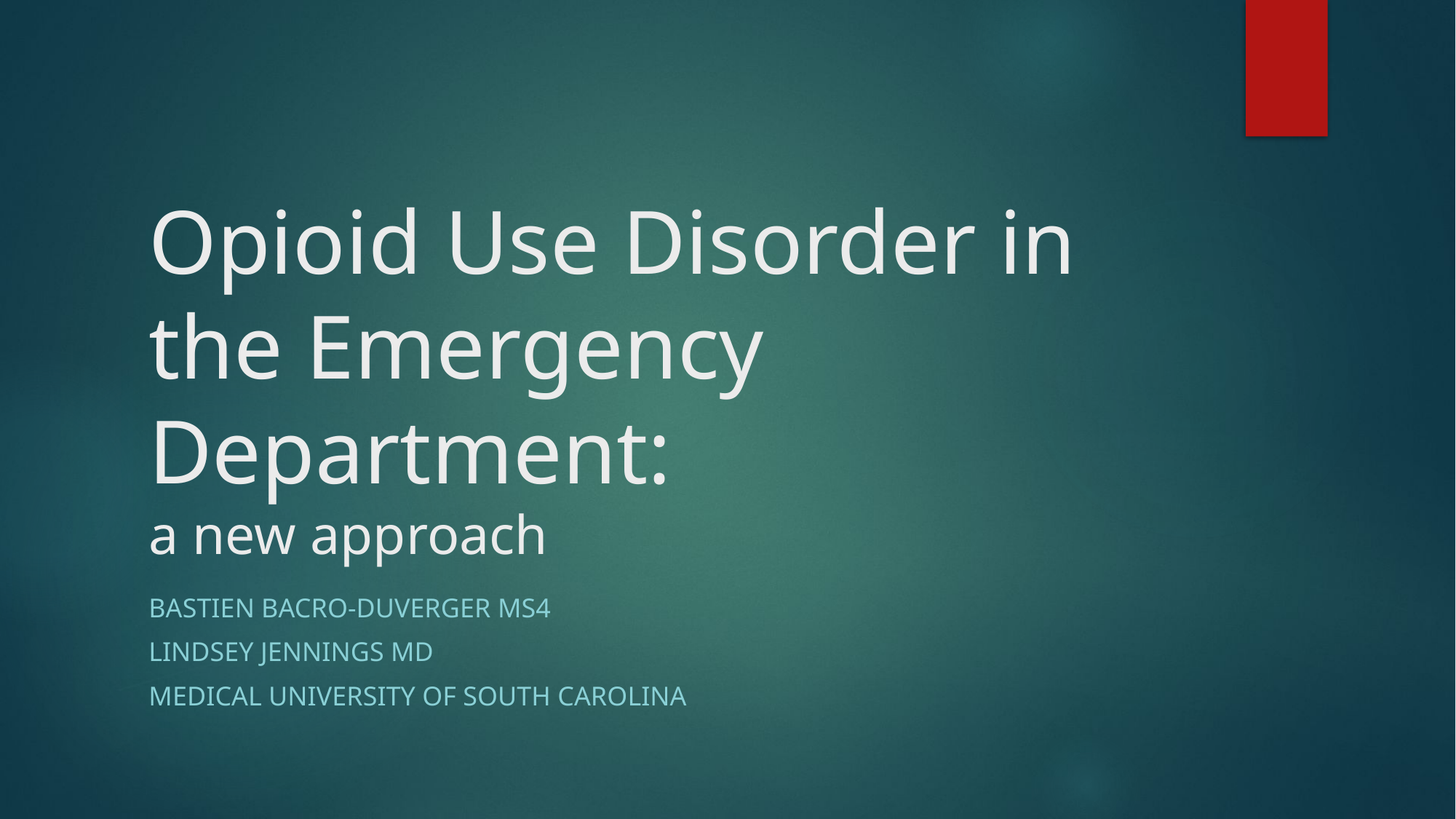

# Opioid Use Disorder in the Emergency Department: a new approach
Bastien Bacro-Duverger Ms4
Lindsey Jennings MD
Medical University of South Carolina

## Slide 2
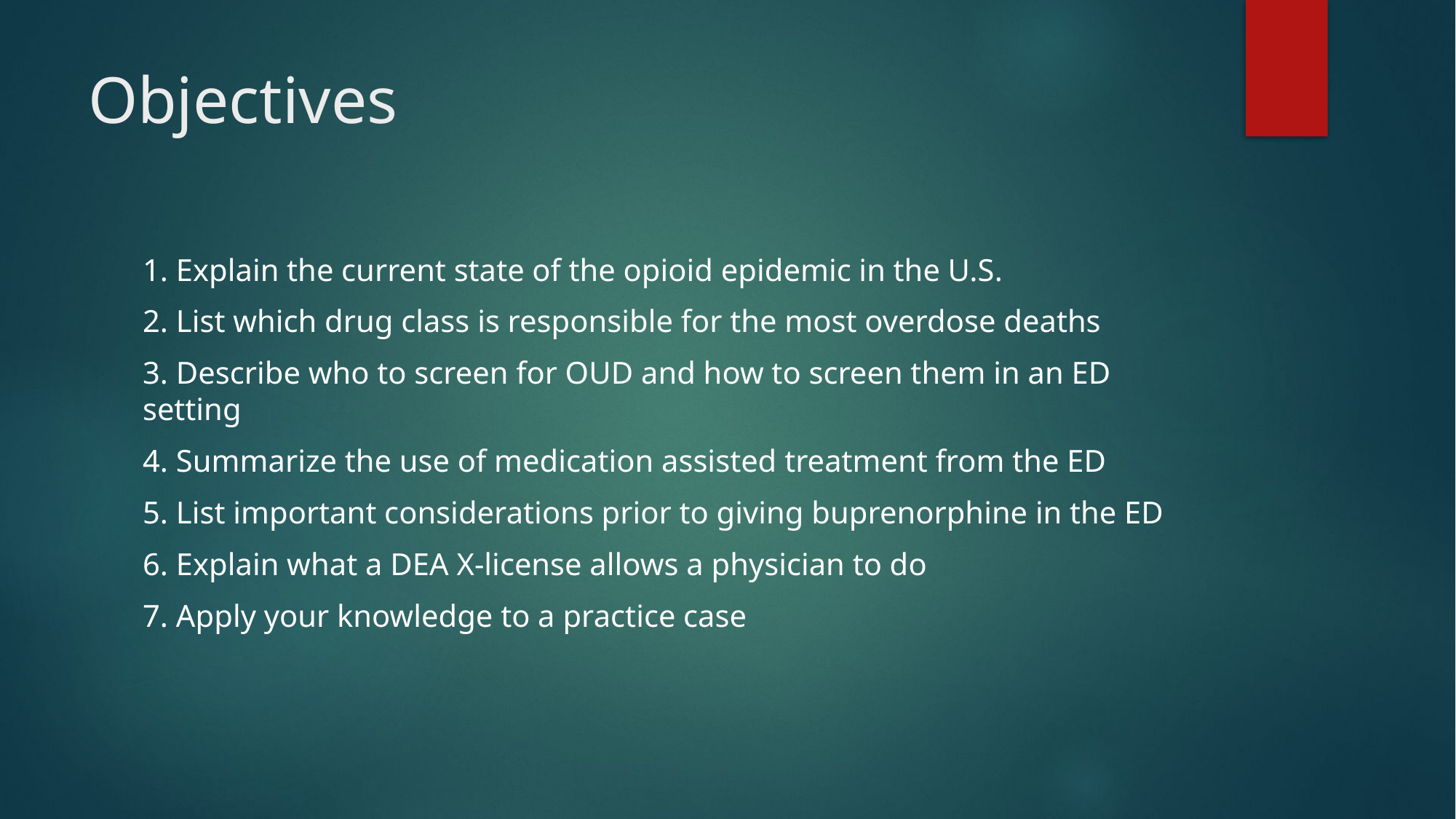

# Objectives
1. Explain the current state of the opioid epidemic in the U.S.
2. List which drug class is responsible for the most overdose deaths
3. Describe who to screen for OUD and how to screen them in an ED setting
4. Summarize the use of medication assisted treatment from the ED
5. List important considerations prior to giving buprenorphine in the ED
6. Explain what a DEA X-license allows a physician to do
7. Apply your knowledge to a practice case

## Slide 3
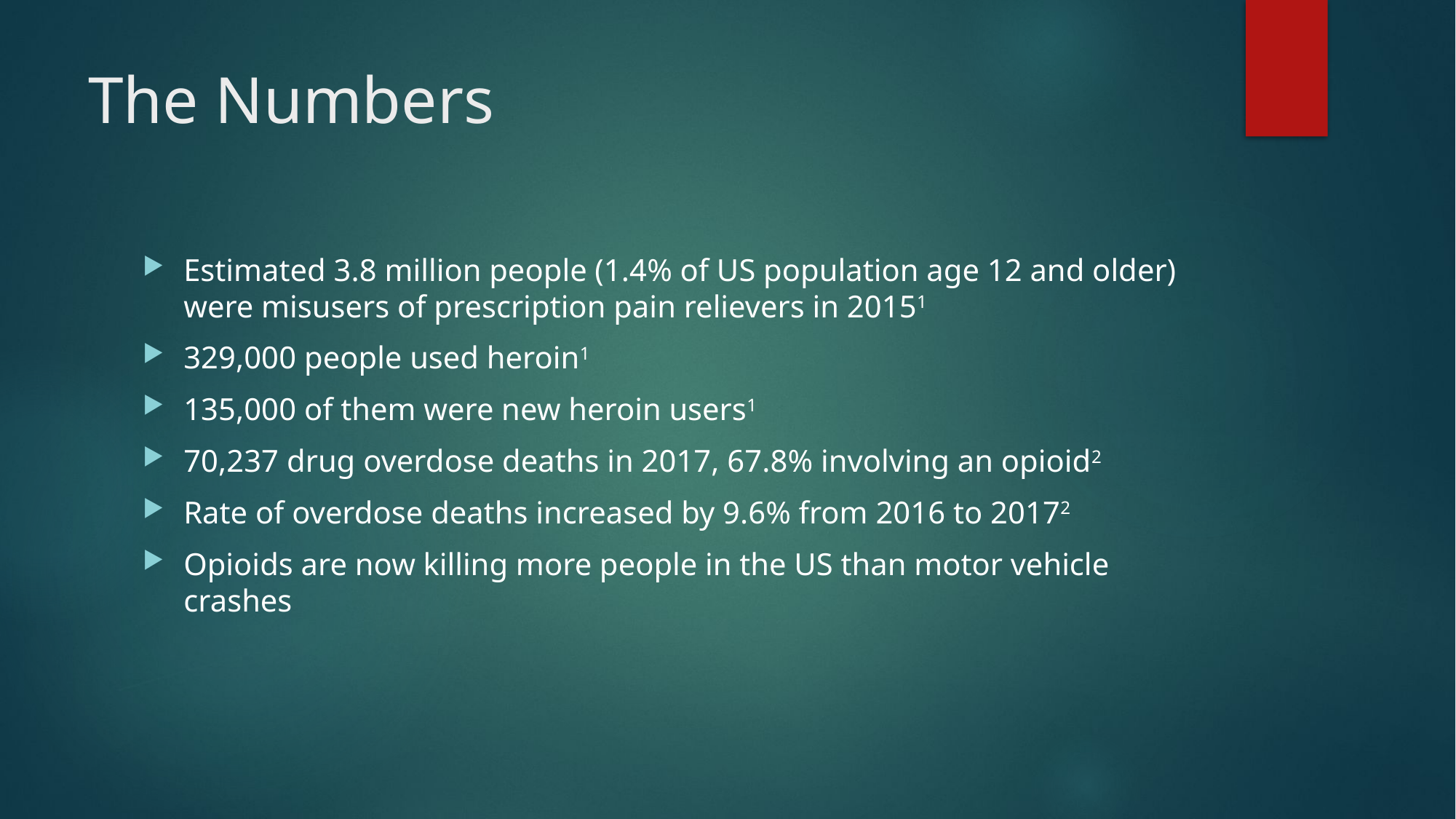

# The Numbers
Estimated 3.8 million people (1.4% of US population age 12 and older) were misusers of prescription pain relievers in 20151
329,000 people used heroin1
135,000 of them were new heroin users1
70,237 drug overdose deaths in 2017, 67.8% involving an opioid2
Rate of overdose deaths increased by 9.6% from 2016 to 20172
Opioids are now killing more people in the US than motor vehicle crashes

## Slide 4
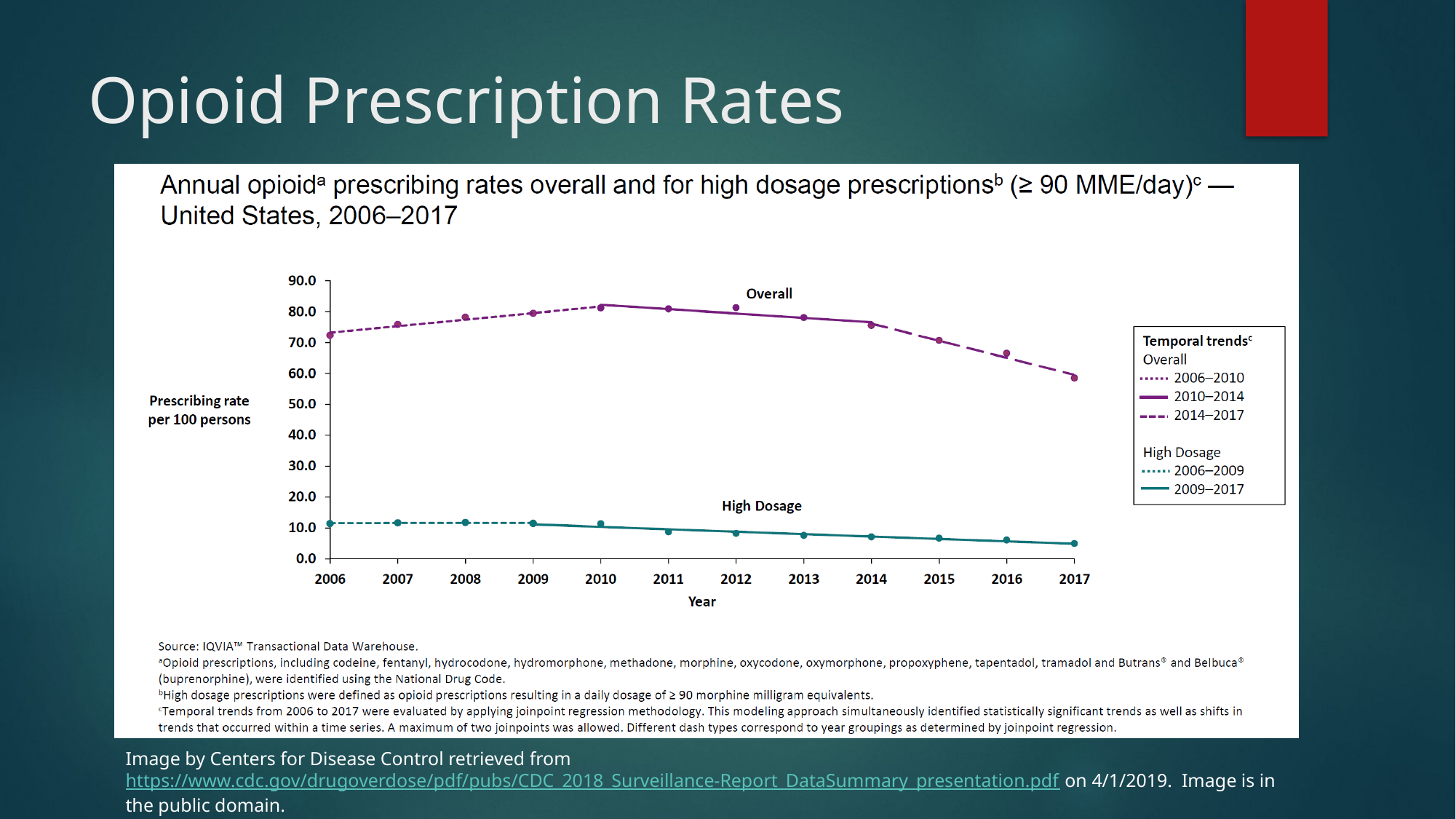

# Opioid Prescription Rates
Image by Centers for Disease Control retrieved from https://www.cdc.gov/drugoverdose/pdf/pubs/CDC_2018_Surveillance-Report_DataSummary_presentation.pdf on 4/1/2019.  Image is in the public domain.

## Slide 5
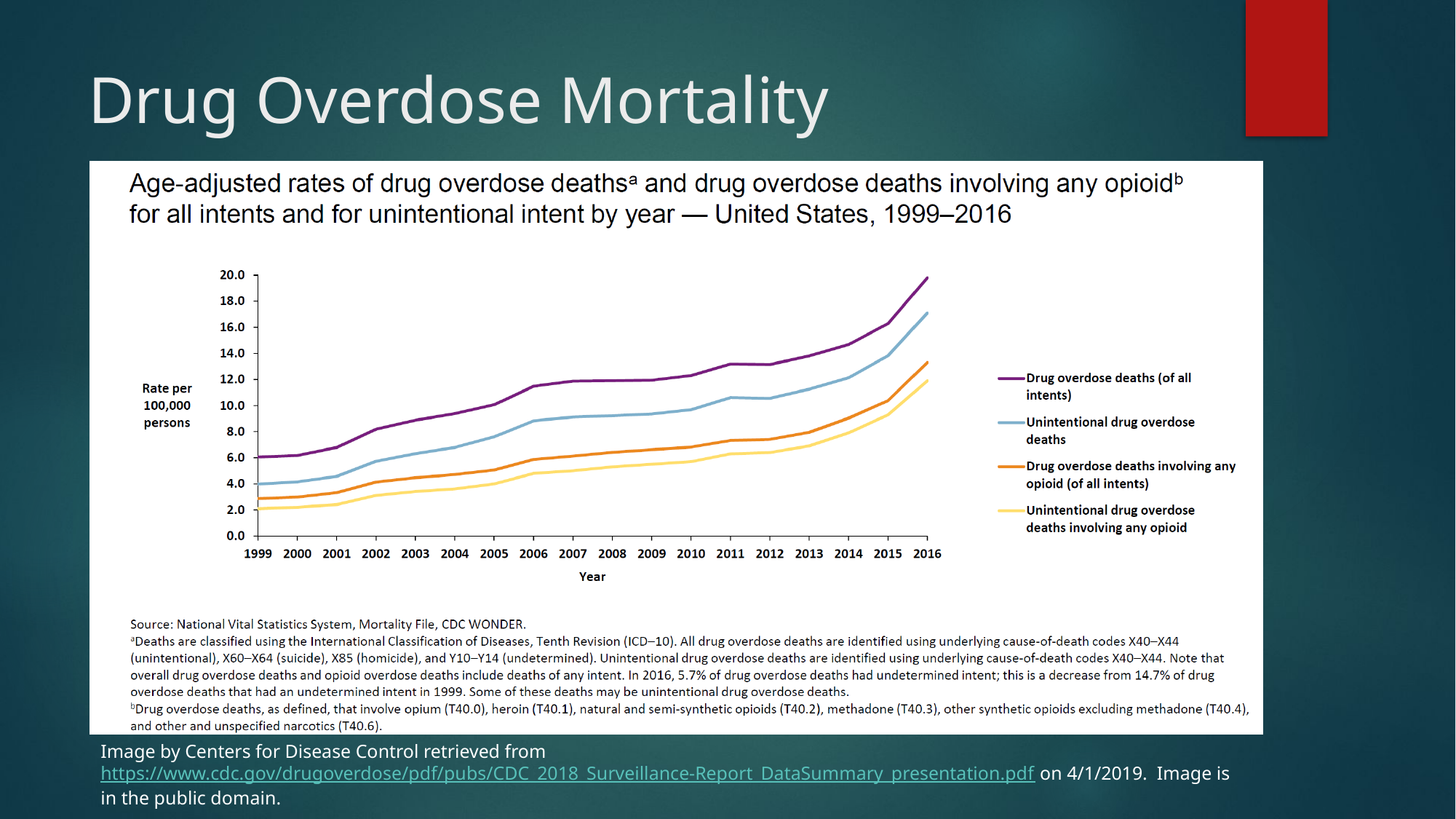

# Drug Overdose Mortality
Image by Centers for Disease Control retrieved from https://www.cdc.gov/drugoverdose/pdf/pubs/CDC_2018_Surveillance-Report_DataSummary_presentation.pdf on 4/1/2019.  Image is in the public domain.

## Slide 6
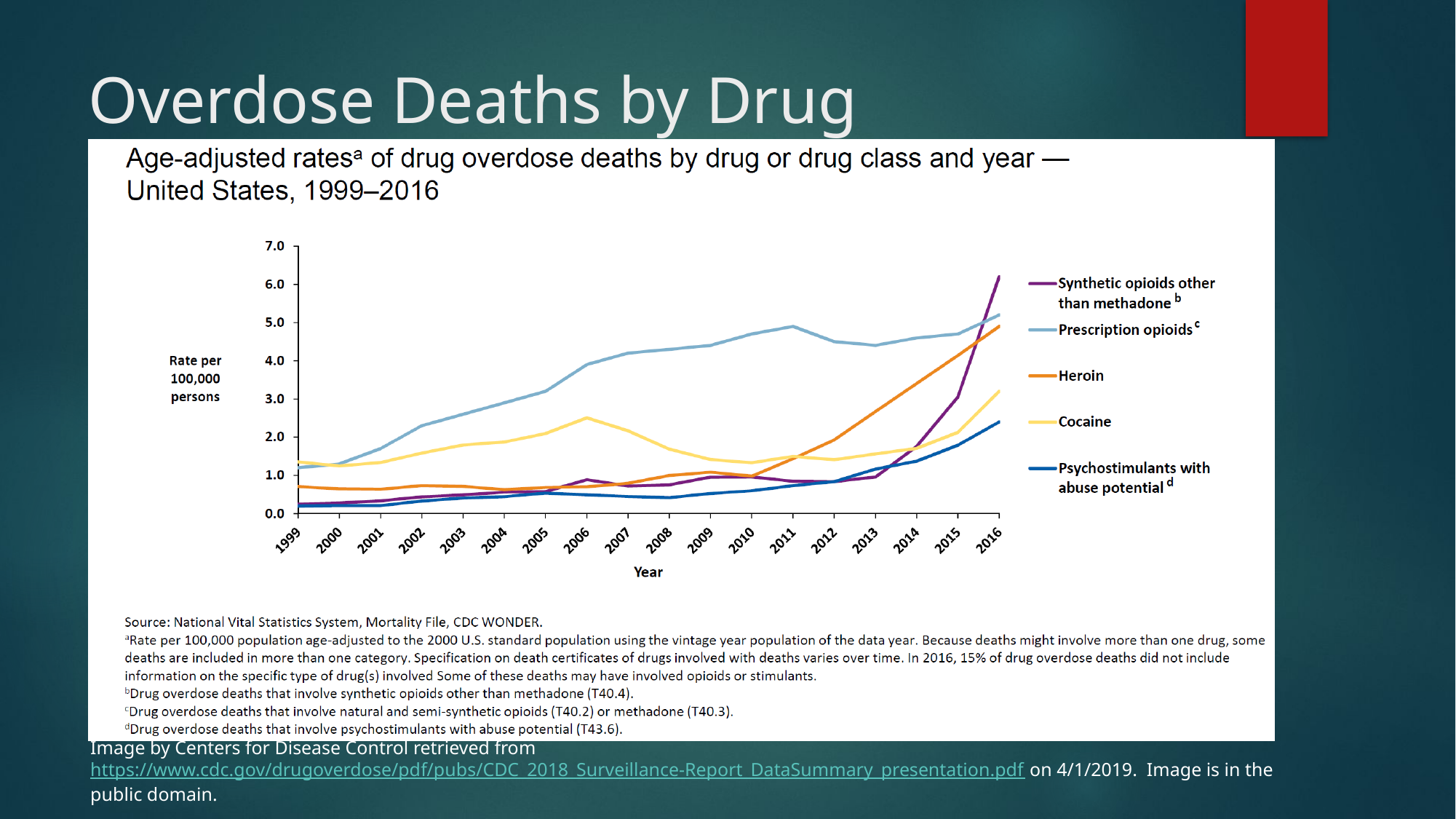

# Overdose Deaths by Drug
Image by Centers for Disease Control retrieved from https://www.cdc.gov/drugoverdose/pdf/pubs/CDC_2018_Surveillance-Report_DataSummary_presentation.pdf on 4/1/2019.  Image is in the public domain.

## Slide 7
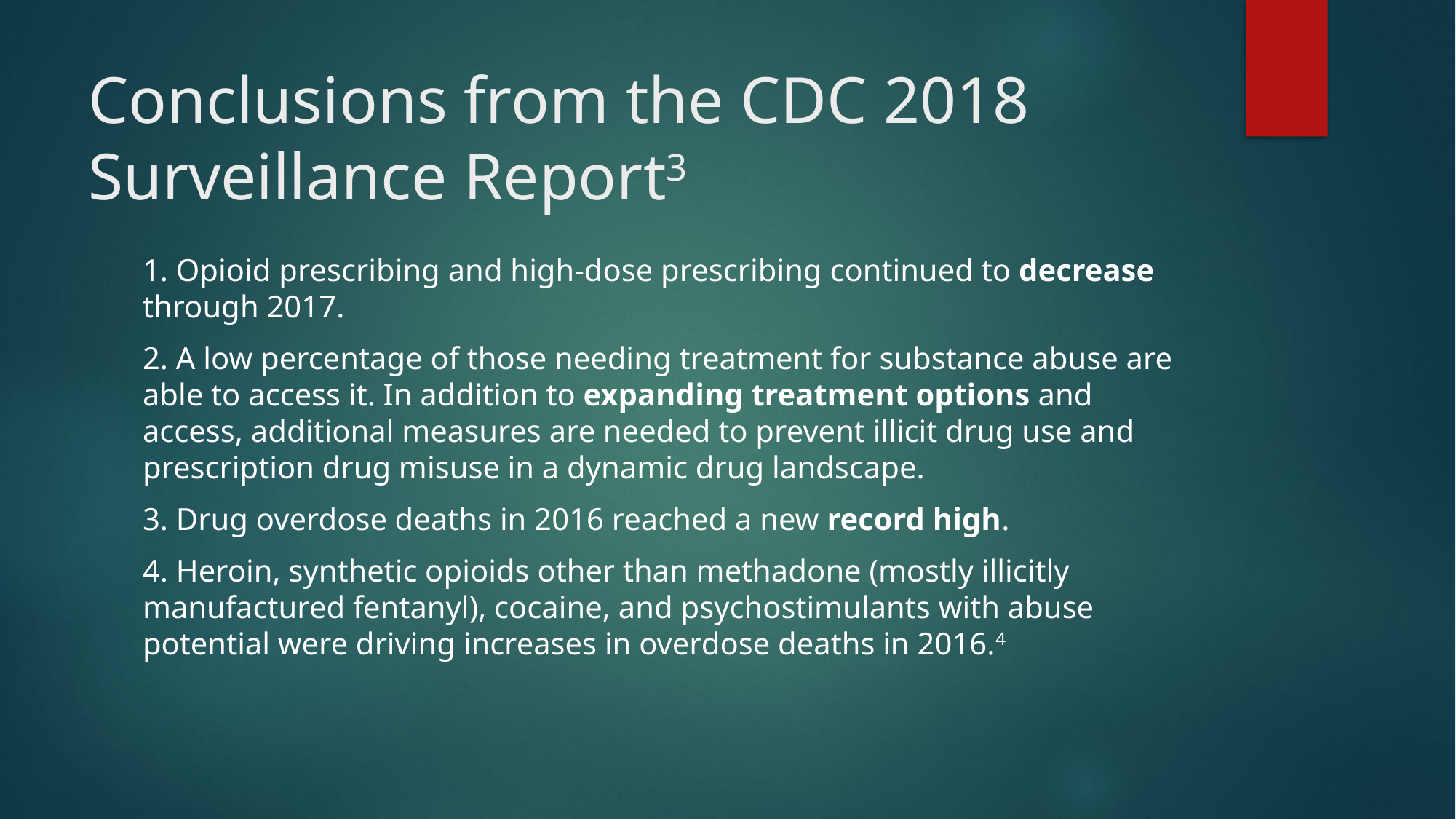

# Conclusions from the CDC 2018 Surveillance Report3
1. Opioid prescribing and high-dose prescribing continued to decrease through 2017.
2. A low percentage of those needing treatment for substance abuse are able to access it. In addition to expanding treatment options and access, additional measures are needed to prevent illicit drug use and prescription drug misuse in a dynamic drug landscape.
3. Drug overdose deaths in 2016 reached a new record high.
4. Heroin, synthetic opioids other than methadone (mostly illicitly manufactured fentanyl), cocaine, and psychostimulants with abuse potential were driving increases in overdose deaths in 2016.4

## Slide 8
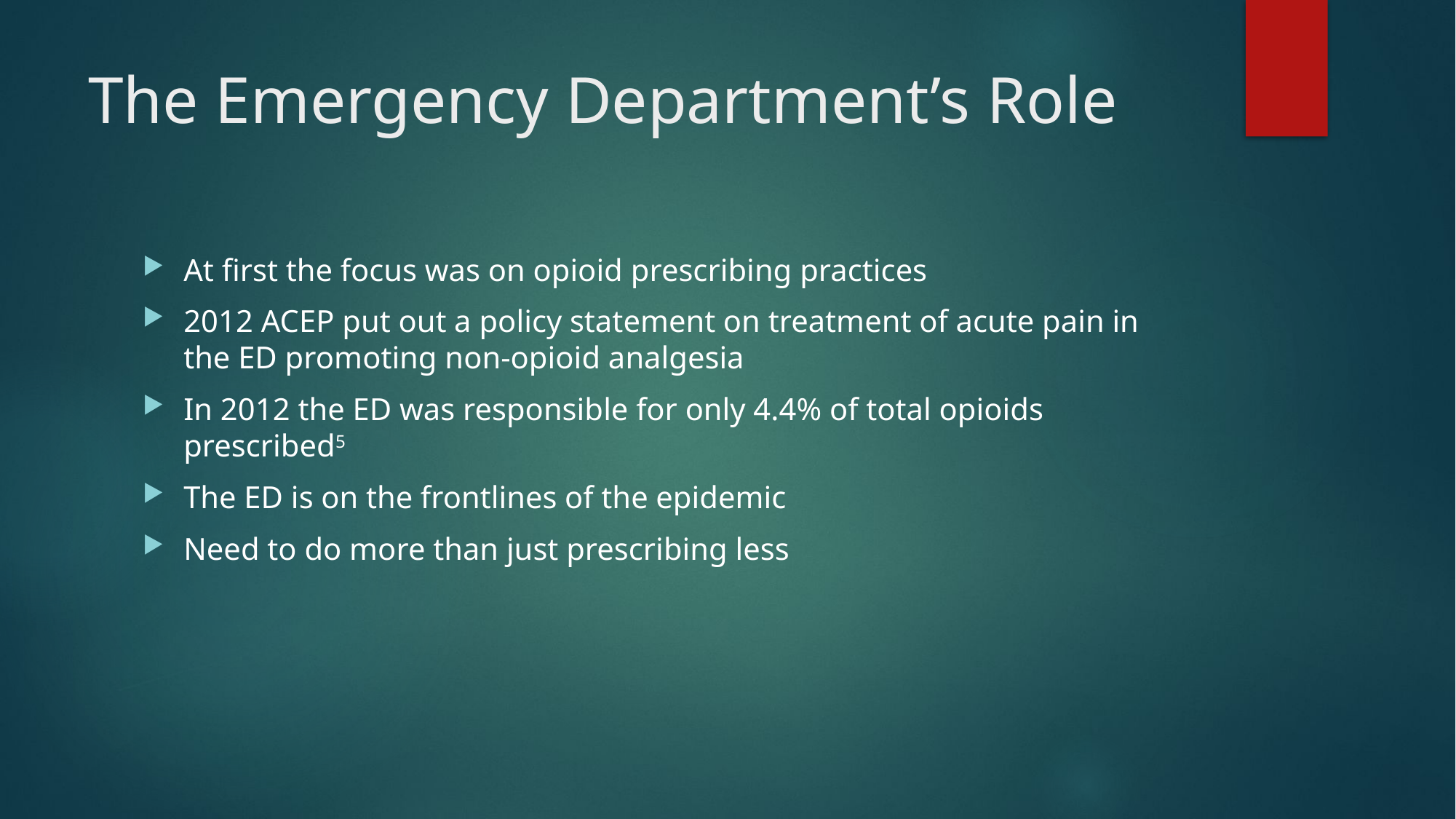

# The Emergency Department’s Role
At first the focus was on opioid prescribing practices
2012 ACEP put out a policy statement on treatment of acute pain in the ED promoting non-opioid analgesia
In 2012 the ED was responsible for only 4.4% of total opioids prescribed5
The ED is on the frontlines of the epidemic
Need to do more than just prescribing less

## Slide 9
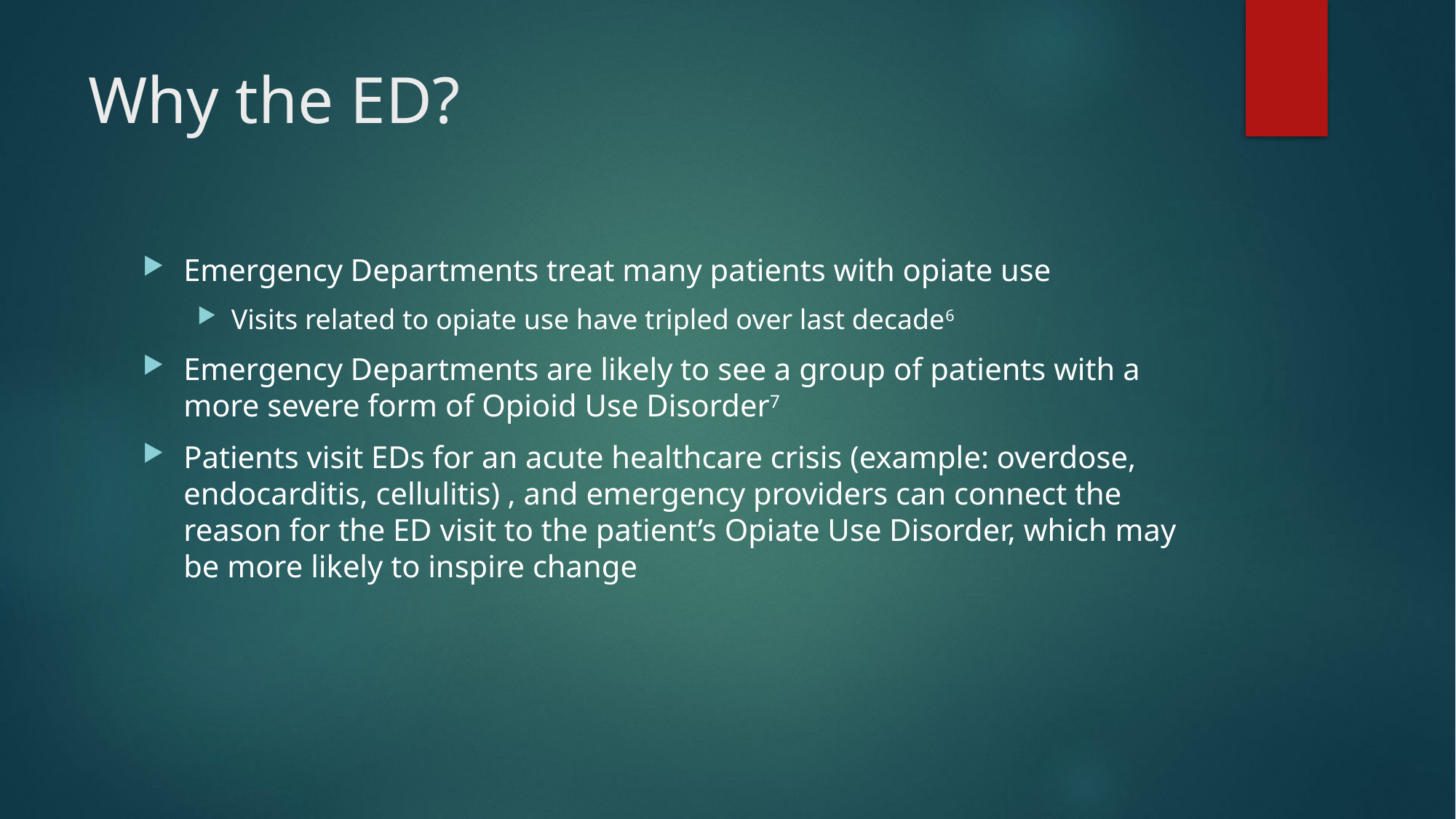

# Why the ED?
Emergency Departments treat many patients with opiate use
Visits related to opiate use have tripled over last decade6
Emergency Departments are likely to see a group of patients with a more severe form of Opioid Use Disorder7
Patients visit EDs for an acute healthcare crisis (example: overdose, endocarditis, cellulitis) , and emergency providers can connect the reason for the ED visit to the patient’s Opiate Use Disorder, which may be more likely to inspire change

## Slide 10
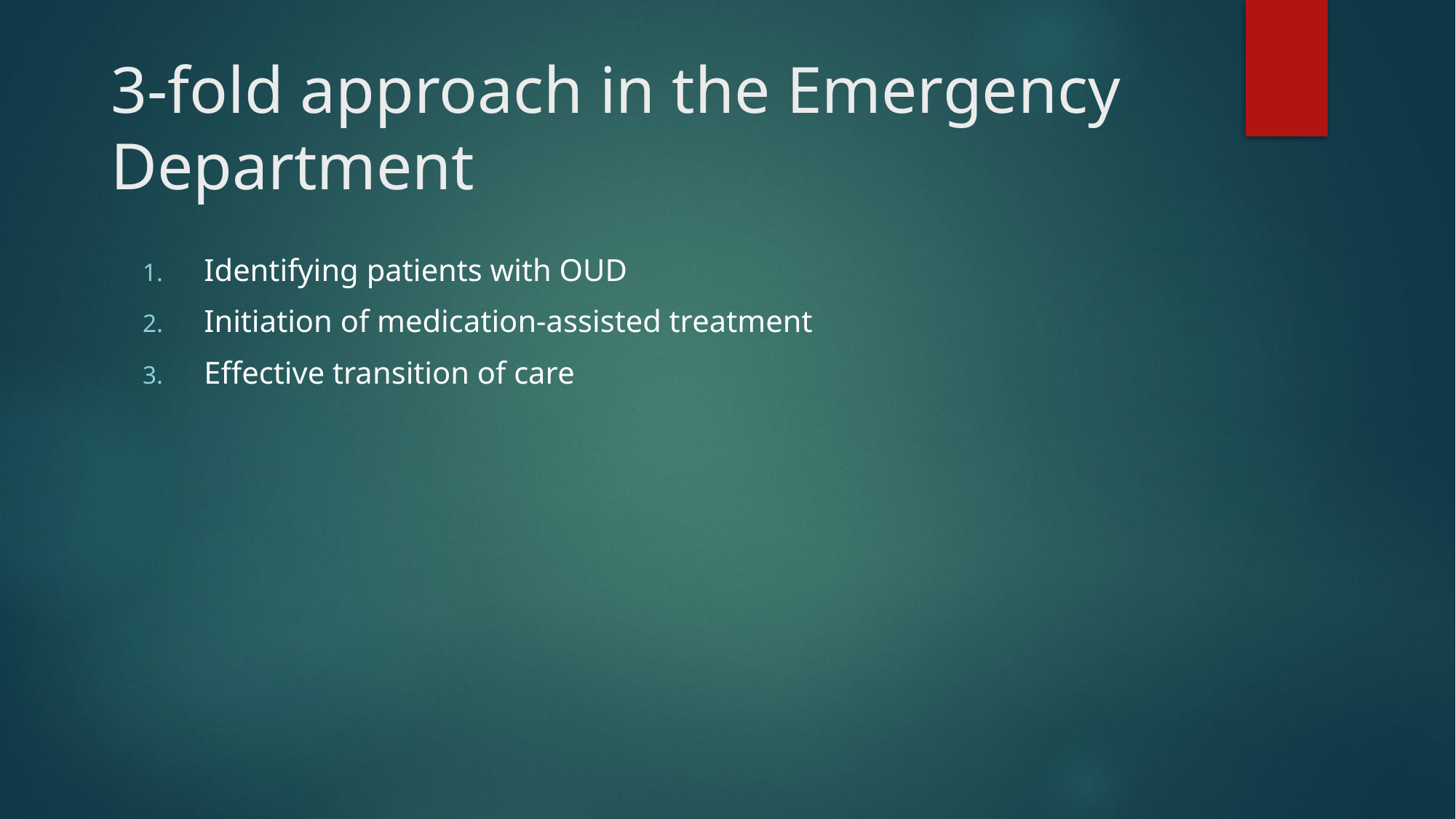

# 3-fold approach in the Emergency Department
Identifying patients with OUD
Initiation of medication-assisted treatment
Effective transition of care

## Slide 11
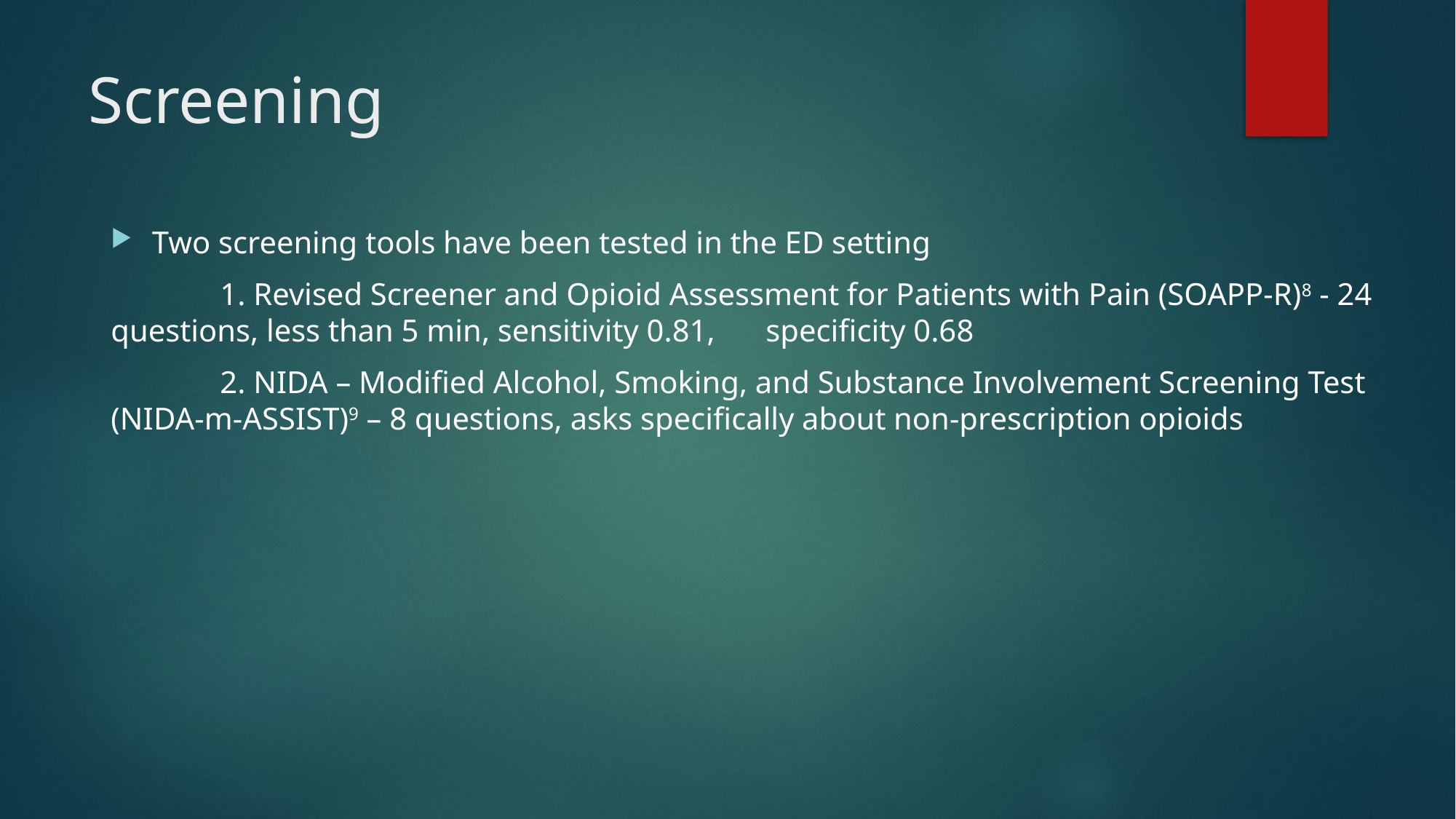

# Screening
Two screening tools have been tested in the ED setting
	1. Revised Screener and Opioid Assessment for Patients with Pain (SOAPP-R)8 - 24 questions, less than 5 min, sensitivity 0.81, 	specificity 0.68
	2. NIDA – Modified Alcohol, Smoking, and Substance Involvement Screening Test (NIDA-m-ASSIST)9 – 8 questions, asks specifically about non-prescription opioids

## Slide 12
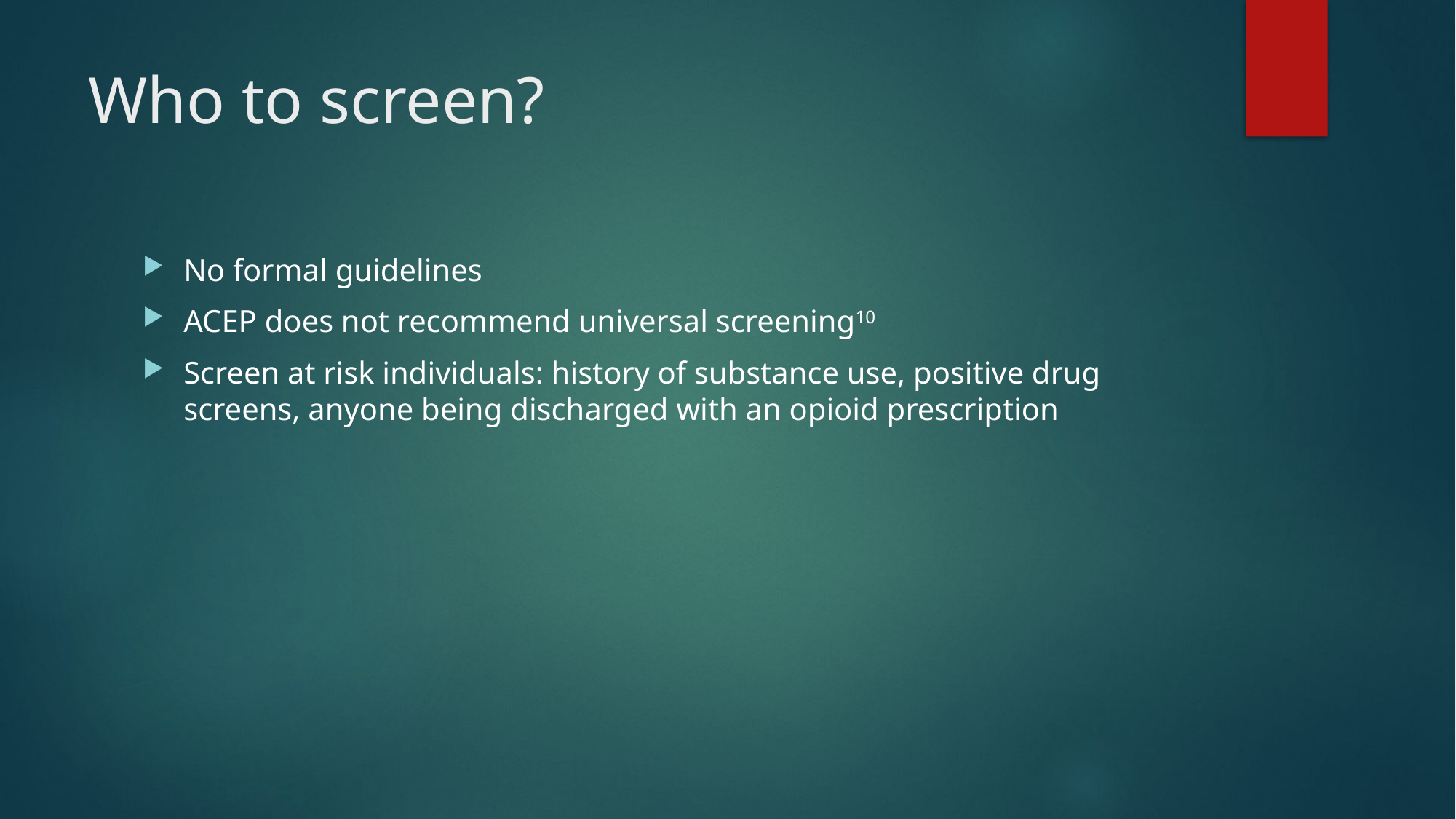

# Who to screen?
No formal guidelines
ACEP does not recommend universal screening10
Screen at risk individuals: history of substance use, positive drug screens, anyone being discharged with an opioid prescription

## Slide 13
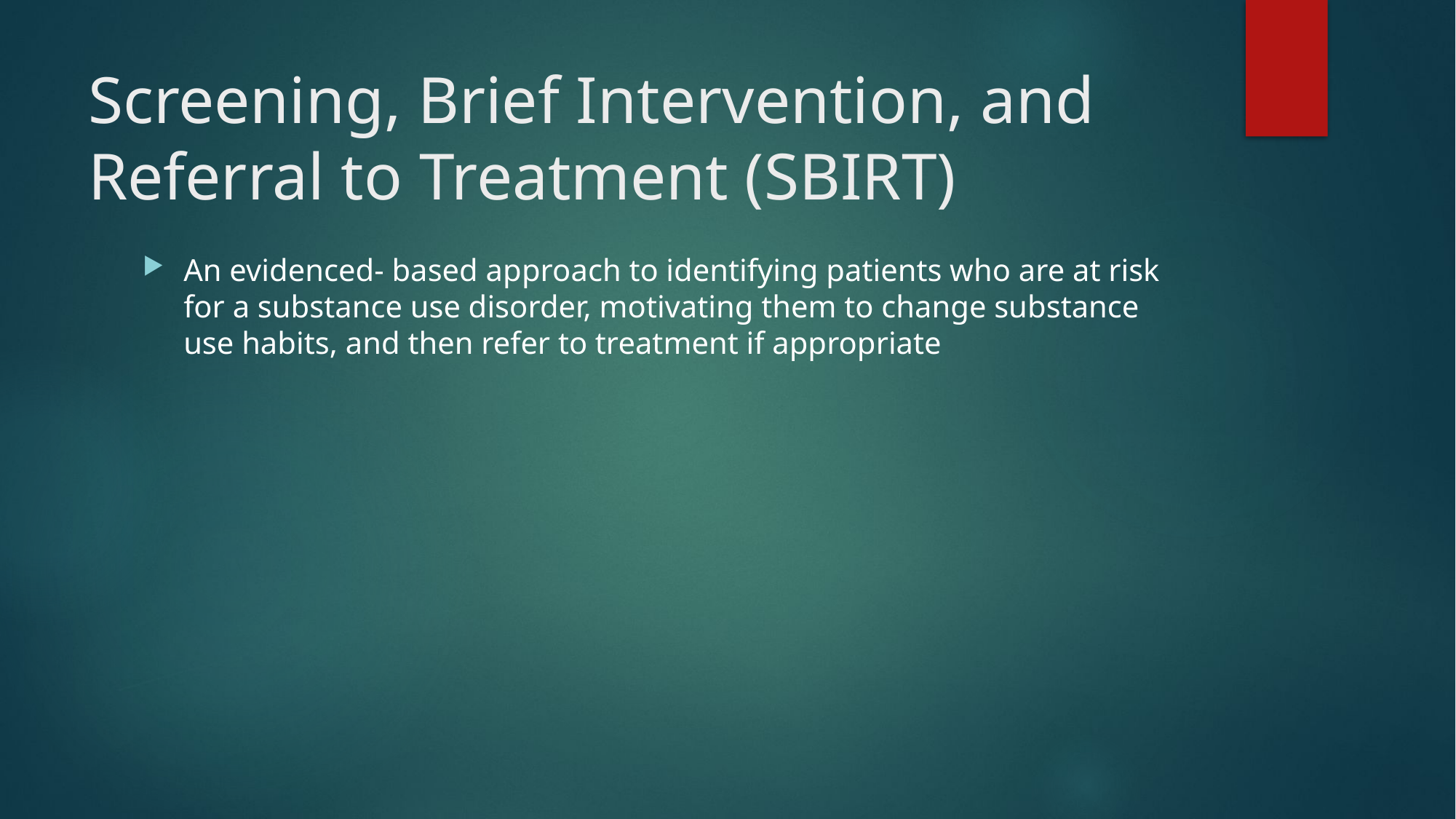

# Screening, Brief Intervention, and Referral to Treatment (SBIRT)
An evidenced- based approach to identifying patients who are at risk for a substance use disorder, motivating them to change substance use habits, and then refer to treatment if appropriate

## Slide 14
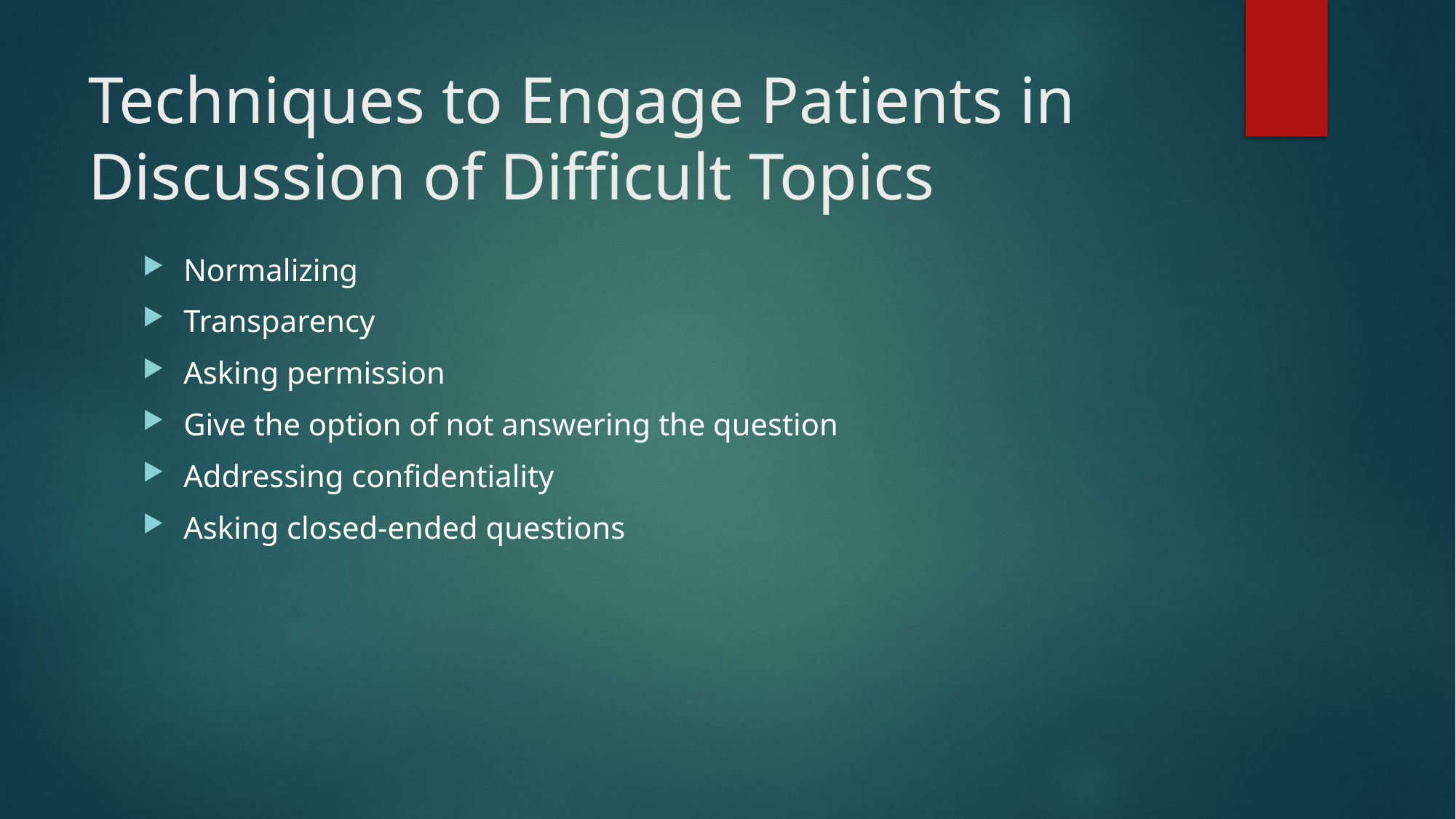

# Techniques to Engage Patients in Discussion of Difficult Topics
Normalizing
Transparency
Asking permission
Give the option of not answering the question
Addressing confidentiality
Asking closed-ended questions

## Slide 15
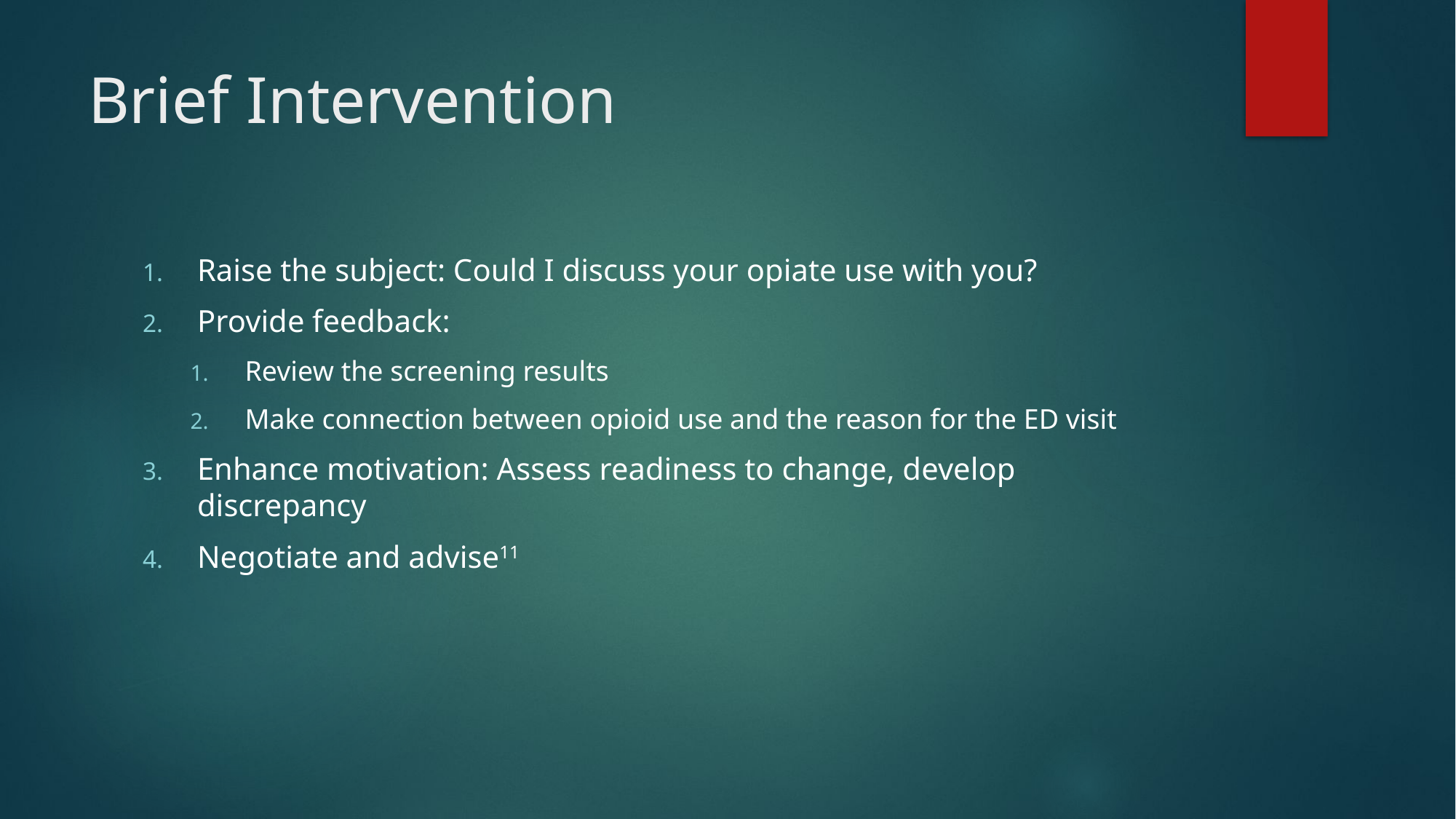

# Brief Intervention
Raise the subject: Could I discuss your opiate use with you?
Provide feedback:
Review the screening results
Make connection between opioid use and the reason for the ED visit
Enhance motivation: Assess readiness to change, develop discrepancy
Negotiate and advise11

## Slide 16
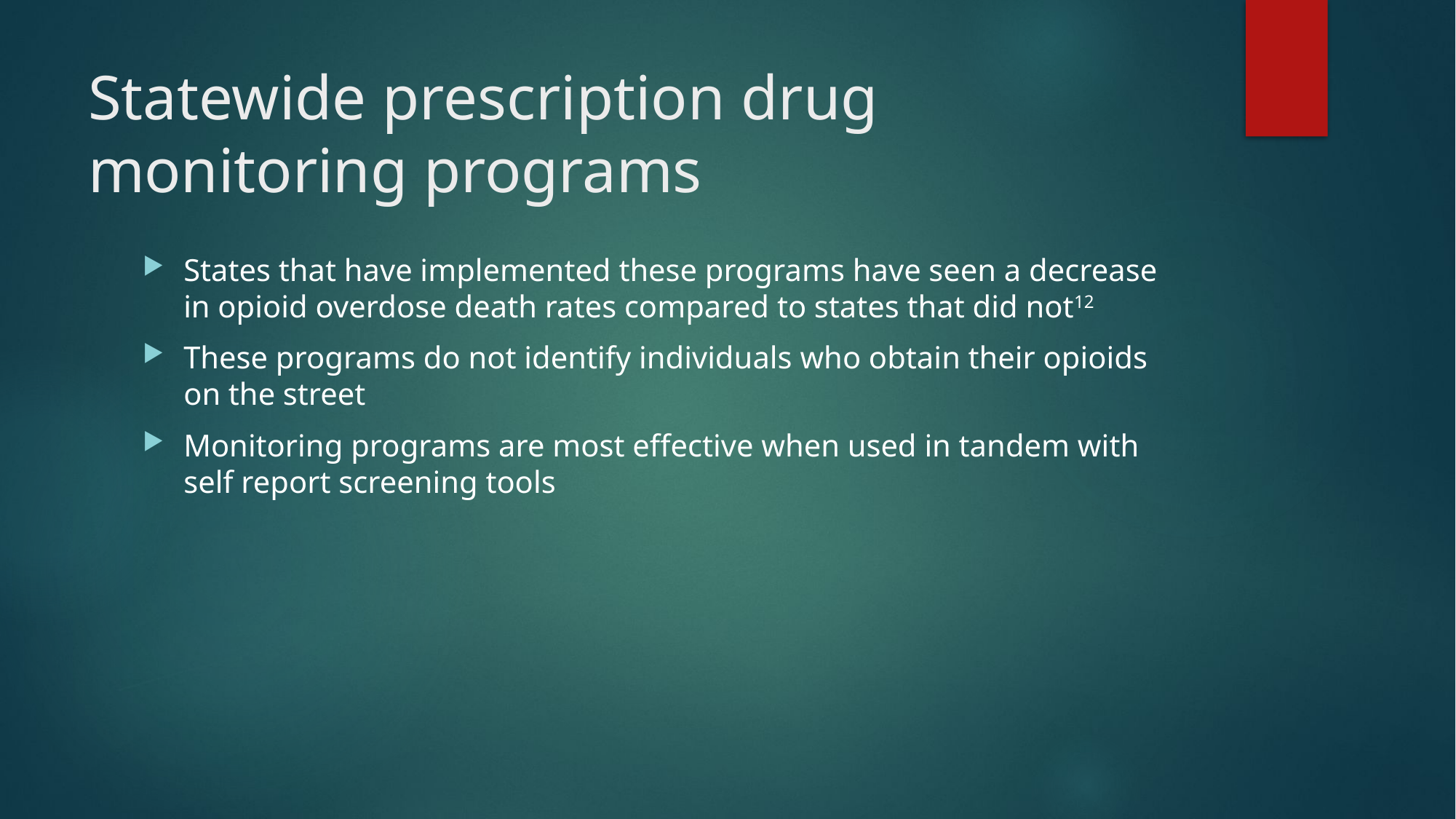

# Statewide prescription drug monitoring programs
States that have implemented these programs have seen a decrease in opioid overdose death rates compared to states that did not12
These programs do not identify individuals who obtain their opioids on the street
Monitoring programs are most effective when used in tandem with self report screening tools

## Slide 17
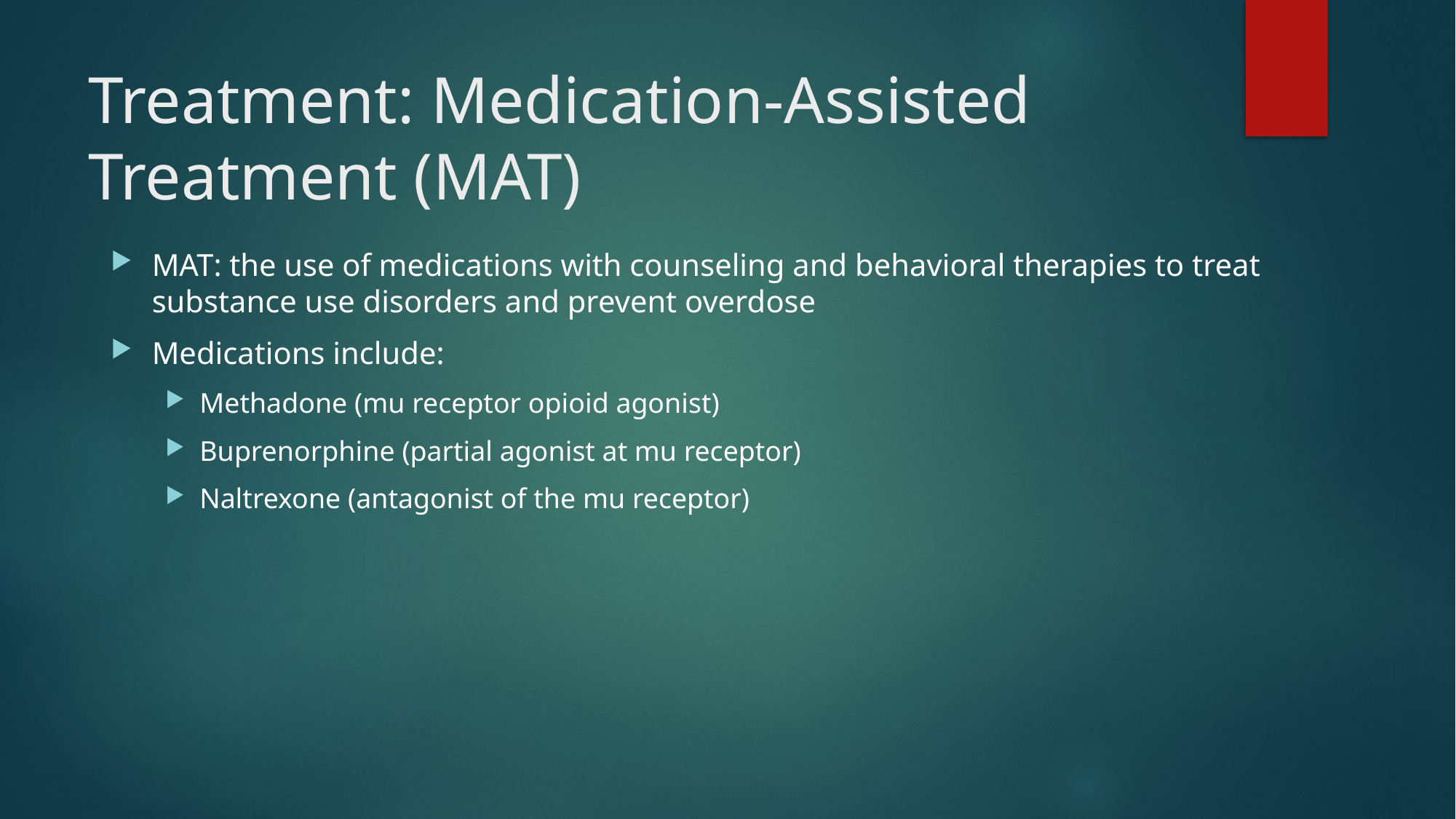

# Treatment: Medication-Assisted Treatment (MAT)
MAT: the use of medications with counseling and behavioral therapies to treat substance use disorders and prevent overdose
Medications include:
Methadone (mu receptor opioid agonist)
Buprenorphine (partial agonist at mu receptor)
Naltrexone (antagonist of the mu receptor)

## Slide 18
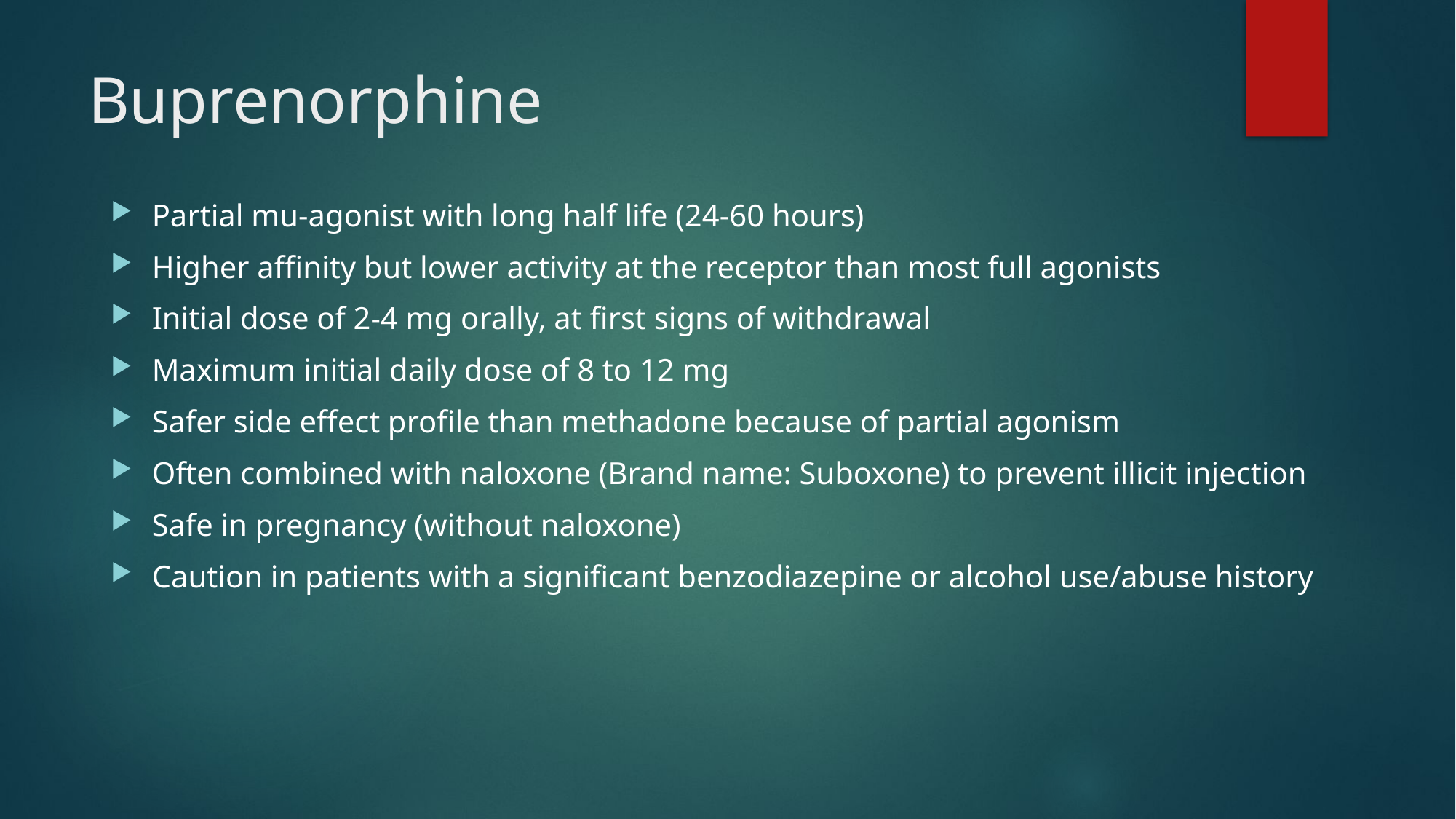

# Buprenorphine
Partial mu-agonist with long half life (24-60 hours)
Higher affinity but lower activity at the receptor than most full agonists
Initial dose of 2-4 mg orally, at first signs of withdrawal
Maximum initial daily dose of 8 to 12 mg
Safer side effect profile than methadone because of partial agonism
Often combined with naloxone (Brand name: Suboxone) to prevent illicit injection
Safe in pregnancy (without naloxone)
Caution in patients with a significant benzodiazepine or alcohol use/abuse history

## Slide 19
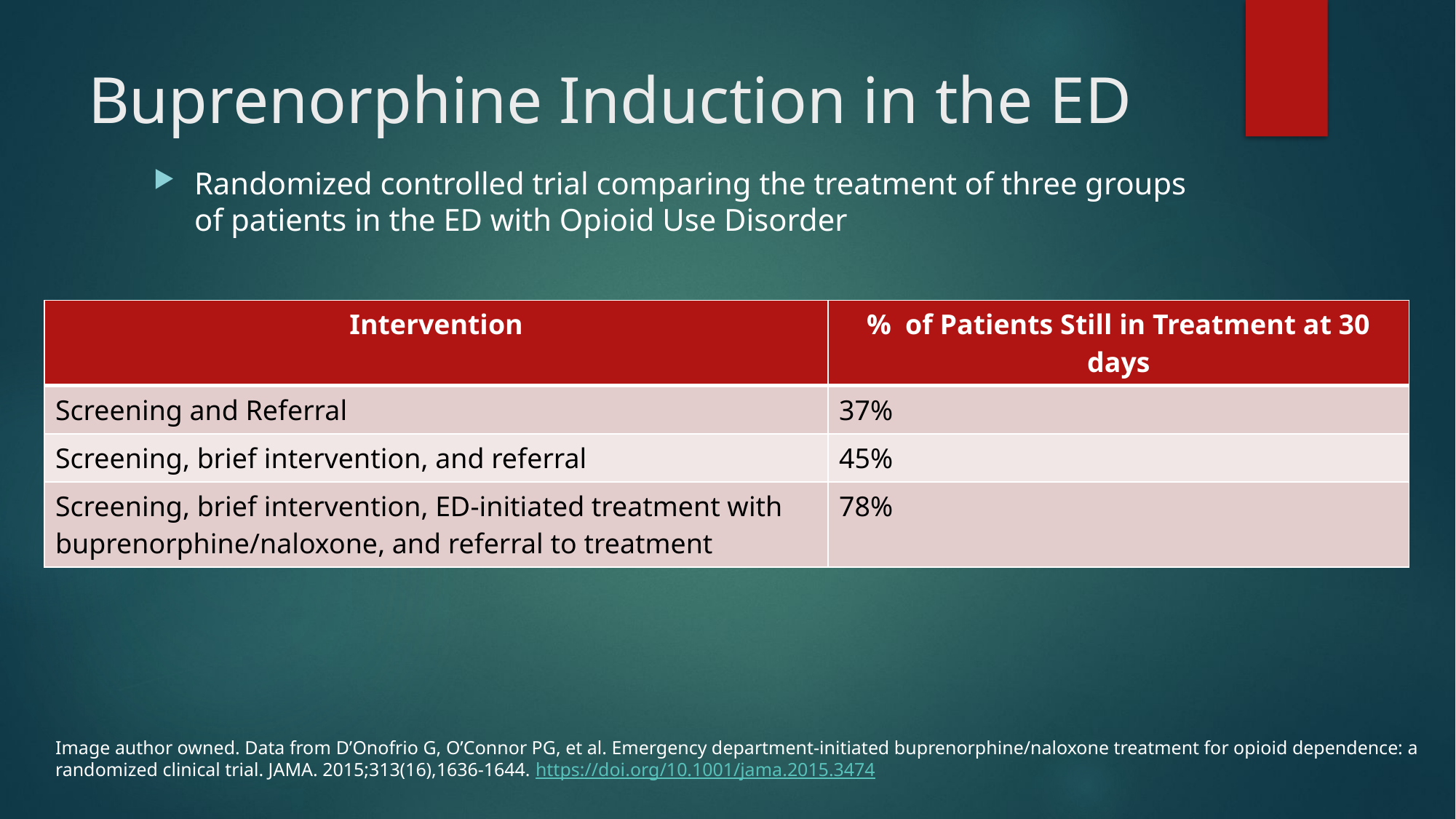

# Buprenorphine Induction in the ED
Randomized controlled trial comparing the treatment of three groups of patients in the ED with Opioid Use Disorder
| Intervention | % of Patients Still in Treatment at 30 days |
| --- | --- |
| Screening and Referral | 37% |
| Screening, brief intervention, and referral | 45% |
| Screening, brief intervention, ED-initiated treatment with buprenorphine/naloxone, and referral to treatment | 78% |
Image author owned. Data from D’Onofrio G, O’Connor PG, et al. Emergency department-initiated buprenorphine/naloxone treatment for opioid dependence: a randomized clinical trial. JAMA. 2015;313(16),1636-1644. https://doi.org/10.1001/jama.2015.3474

## Slide 20
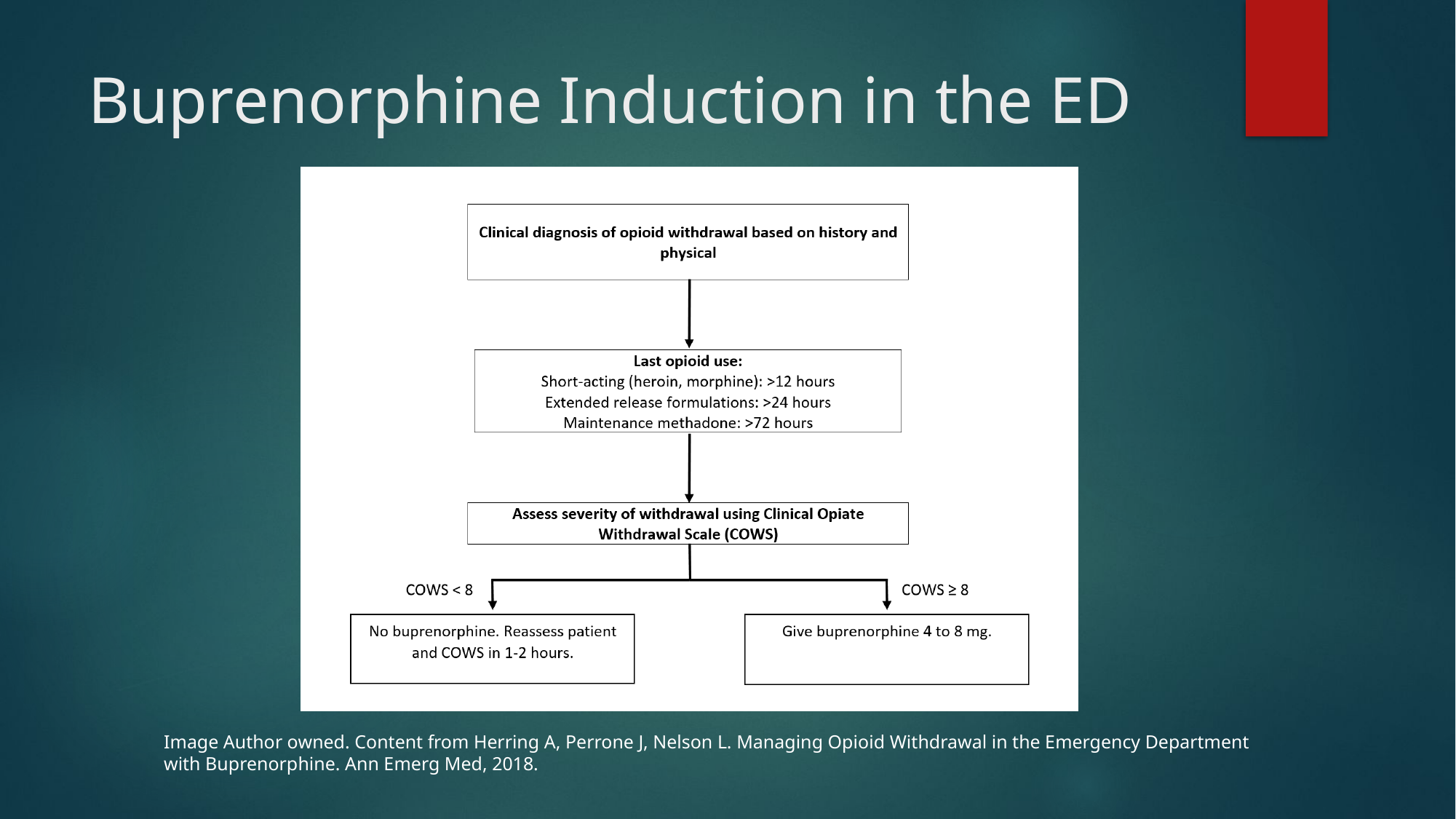

# Buprenorphine Induction in the ED
Image Author owned. Content from Herring A, Perrone J, Nelson L. Managing Opioid Withdrawal in the Emergency Department with Buprenorphine. Ann Emerg Med, 2018.

## Slide 21
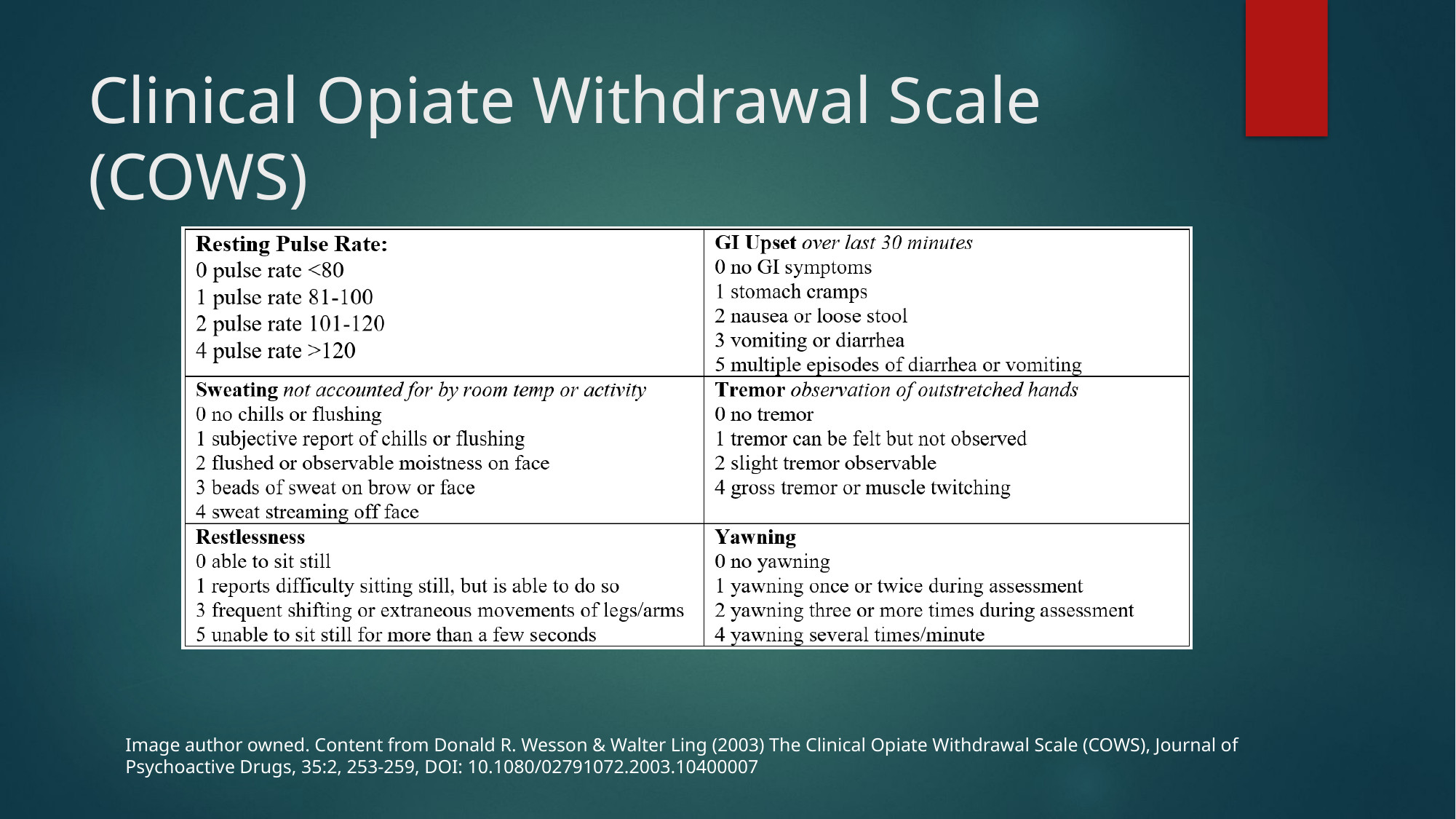

# Clinical Opiate Withdrawal Scale (COWS)
Image author owned. Content from Donald R. Wesson & Walter Ling (2003) The Clinical Opiate Withdrawal Scale (COWS), Journal of Psychoactive Drugs, 35:2, 253-259, DOI: 10.1080/02791072.2003.10400007

## Slide 22
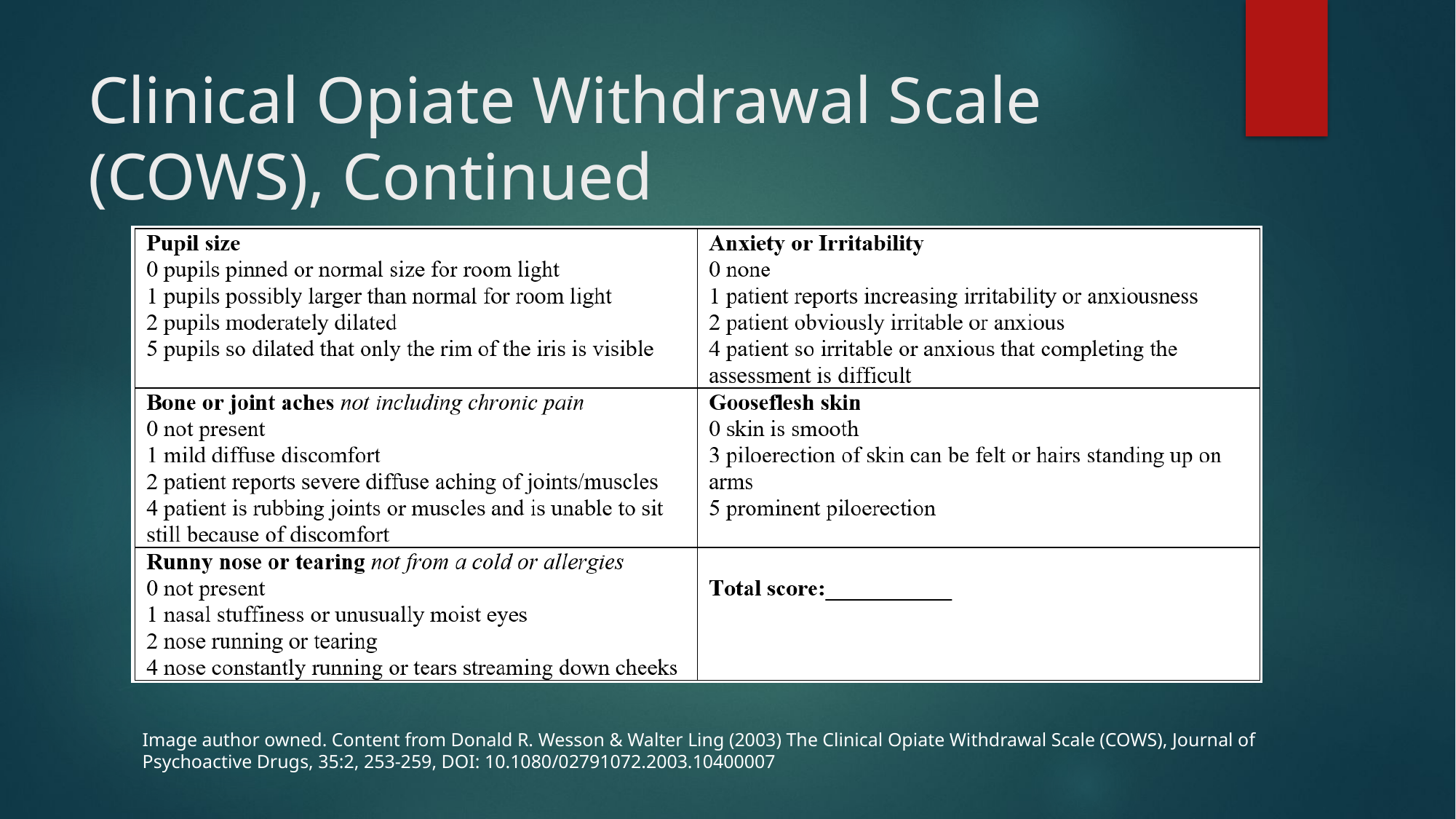

# Clinical Opiate Withdrawal Scale (COWS), Continued
Image author owned. Content from Donald R. Wesson & Walter Ling (2003) The Clinical Opiate Withdrawal Scale (COWS), Journal of Psychoactive Drugs, 35:2, 253-259, DOI: 10.1080/02791072.2003.10400007

## Slide 23
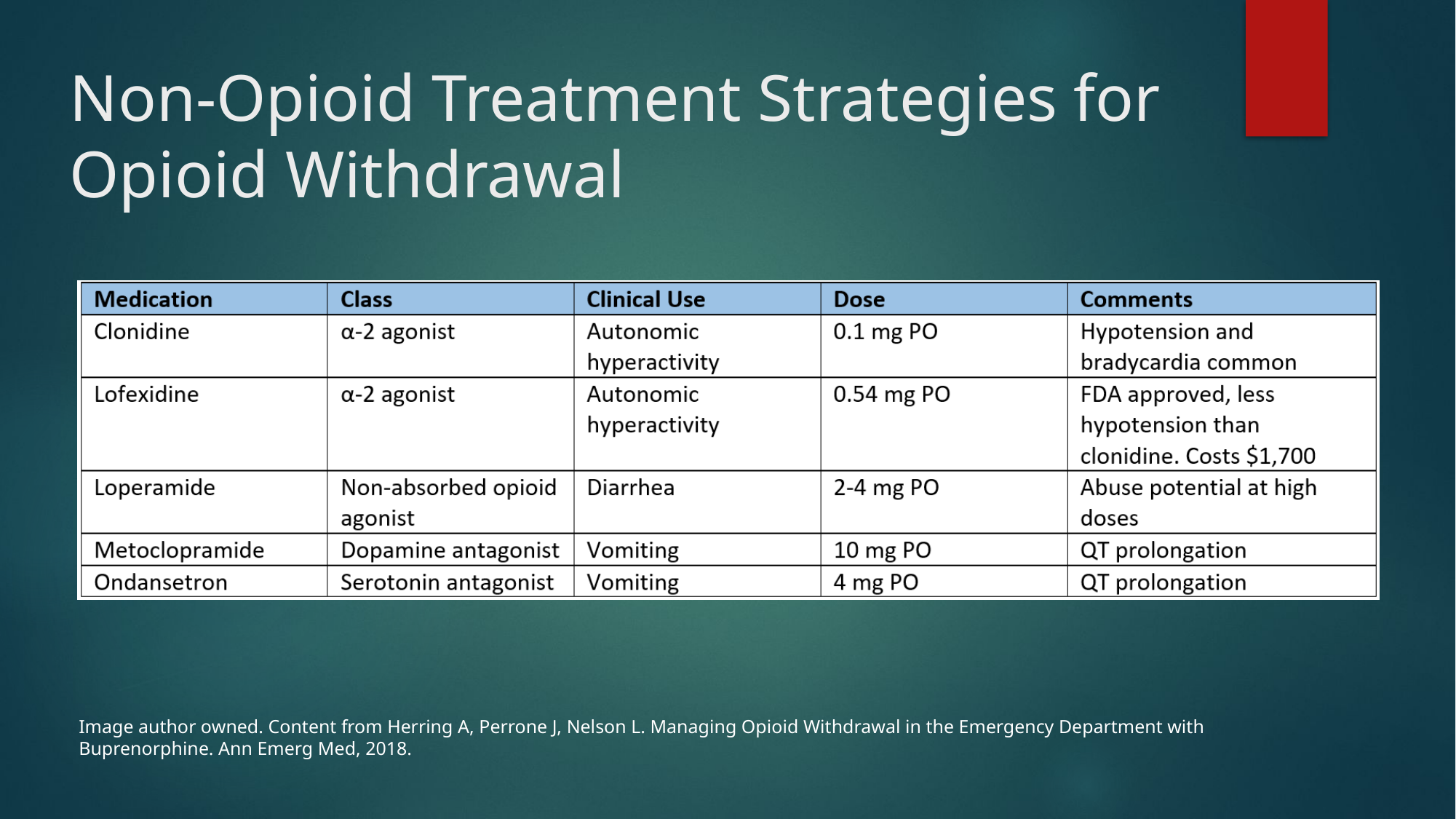

# Non-Opioid Treatment Strategies for Opioid Withdrawal
Image author owned. Content from Herring A, Perrone J, Nelson L. Managing Opioid Withdrawal in the Emergency Department with Buprenorphine. Ann Emerg Med, 2018.

## Slide 24
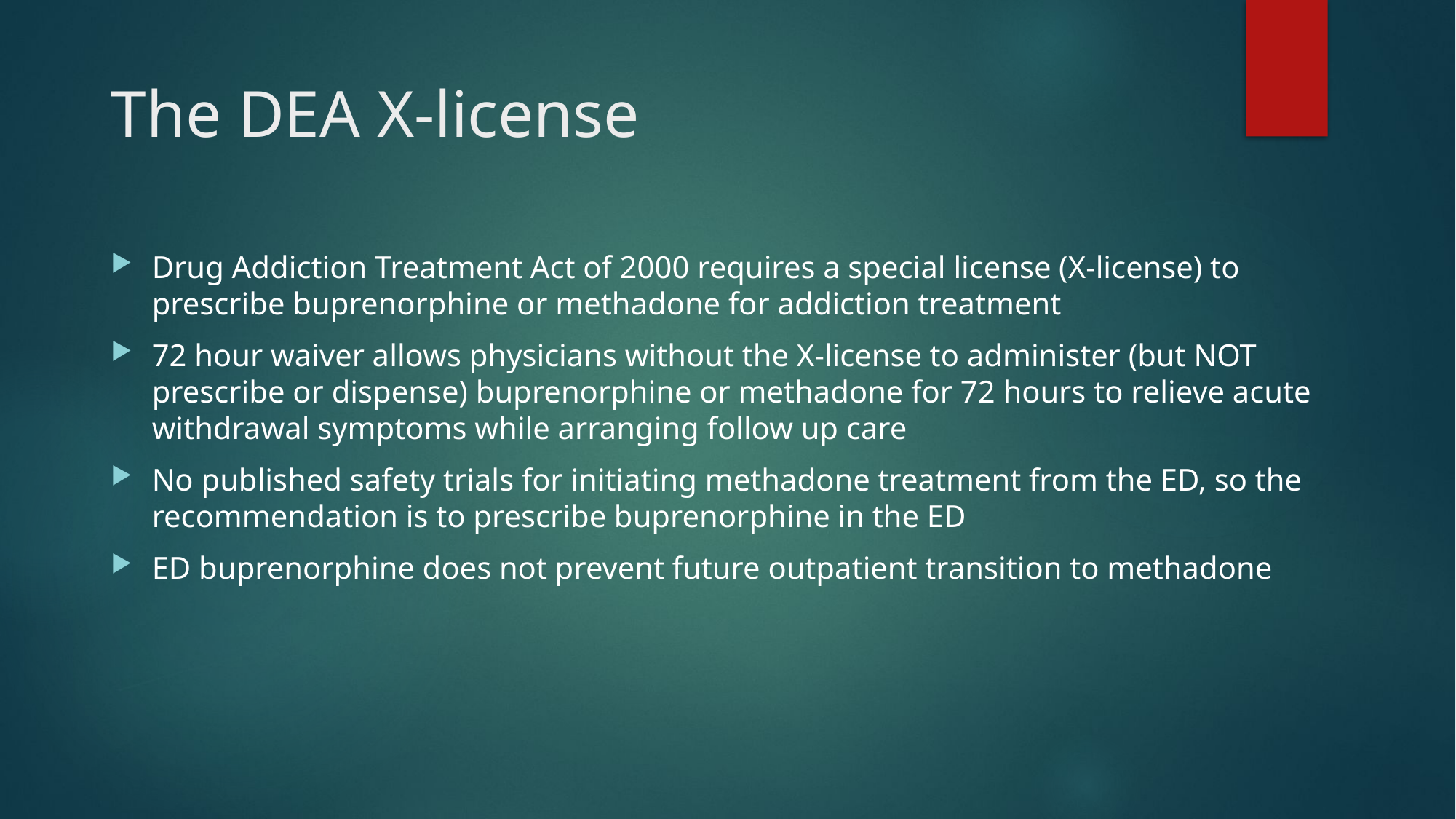

# The DEA X-license
Drug Addiction Treatment Act of 2000 requires a special license (X-license) to prescribe buprenorphine or methadone for addiction treatment
72 hour waiver allows physicians without the X-license to administer (but NOT prescribe or dispense) buprenorphine or methadone for 72 hours to relieve acute withdrawal symptoms while arranging follow up care
No published safety trials for initiating methadone treatment from the ED, so the recommendation is to prescribe buprenorphine in the ED
ED buprenorphine does not prevent future outpatient transition to methadone

## Slide 25
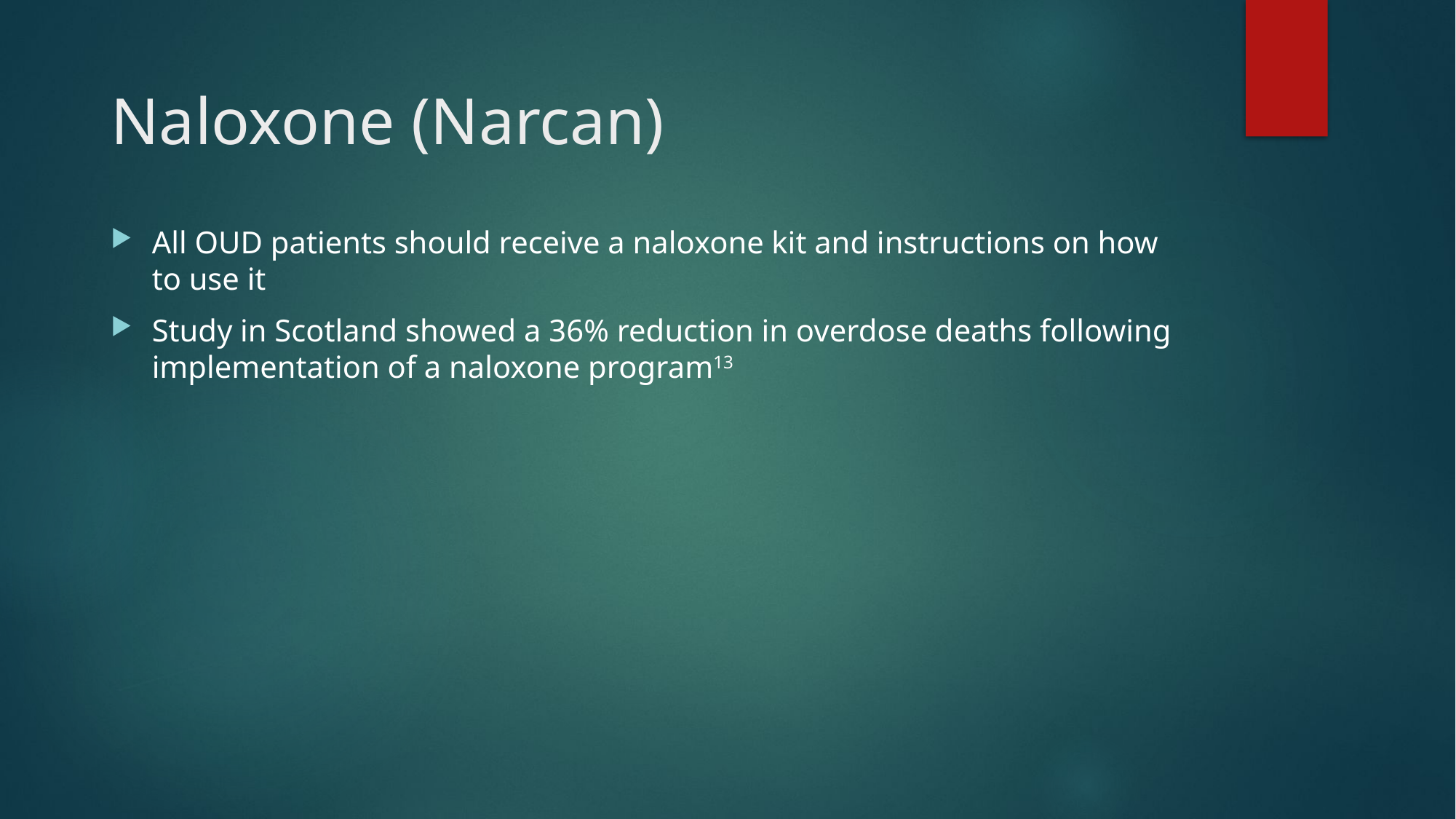

# Naloxone (Narcan)
All OUD patients should receive a naloxone kit and instructions on how to use it
Study in Scotland showed a 36% reduction in overdose deaths following implementation of a naloxone program13

## Slide 26
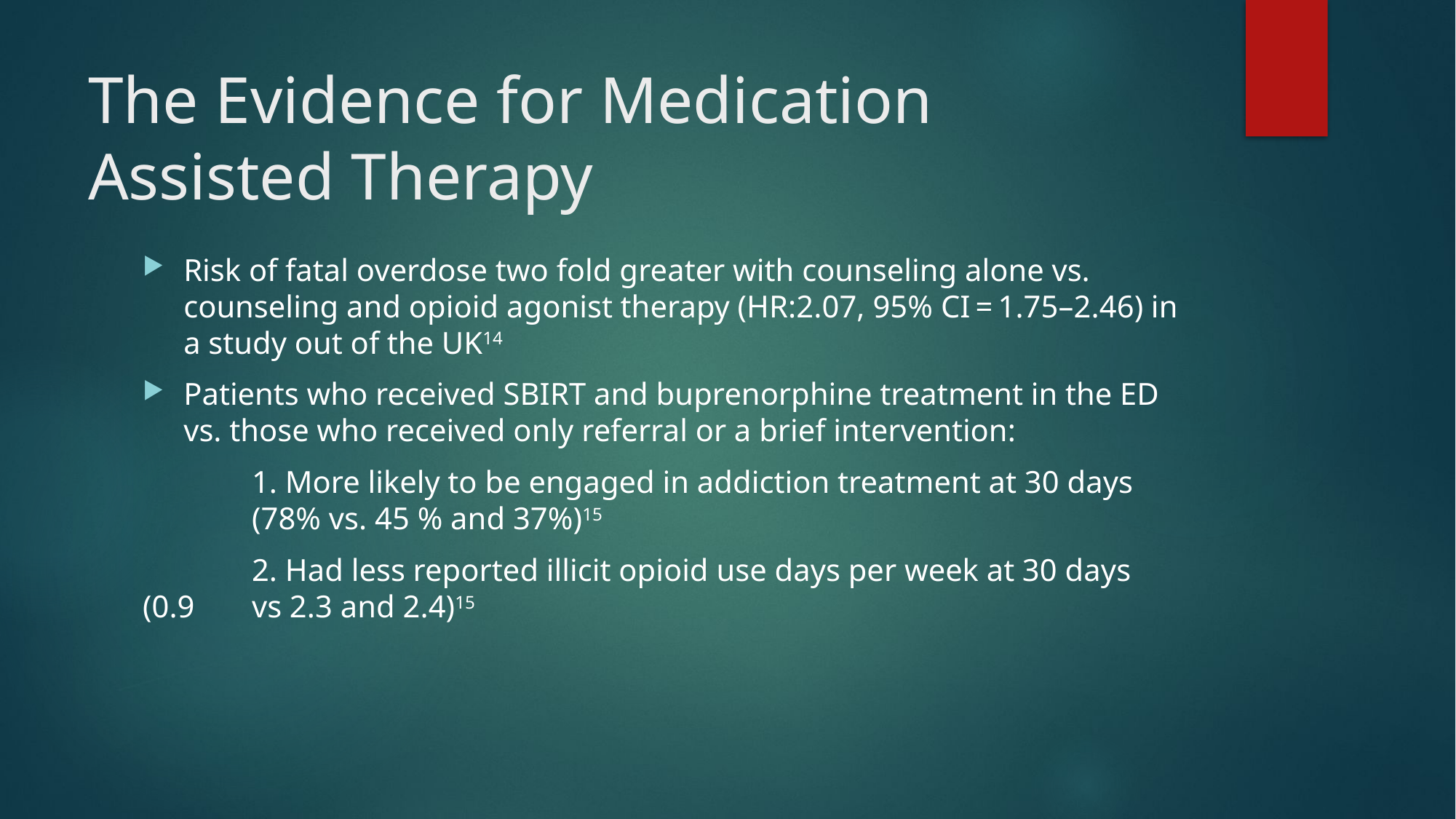

# The Evidence for Medication Assisted Therapy
Risk of fatal overdose two fold greater with counseling alone vs. counseling and opioid agonist therapy (HR:2.07, 95% CI = 1.75–2.46) in a study out of the UK14
Patients who received SBIRT and buprenorphine treatment in the ED vs. those who received only referral or a brief intervention:
	1. More likely to be engaged in addiction treatment at 30 days 	(78% vs. 45 % and 37%)15
	2. Had less reported illicit opioid use days per week at 30 days (0.9 	vs 2.3 and 2.4)15

## Slide 27
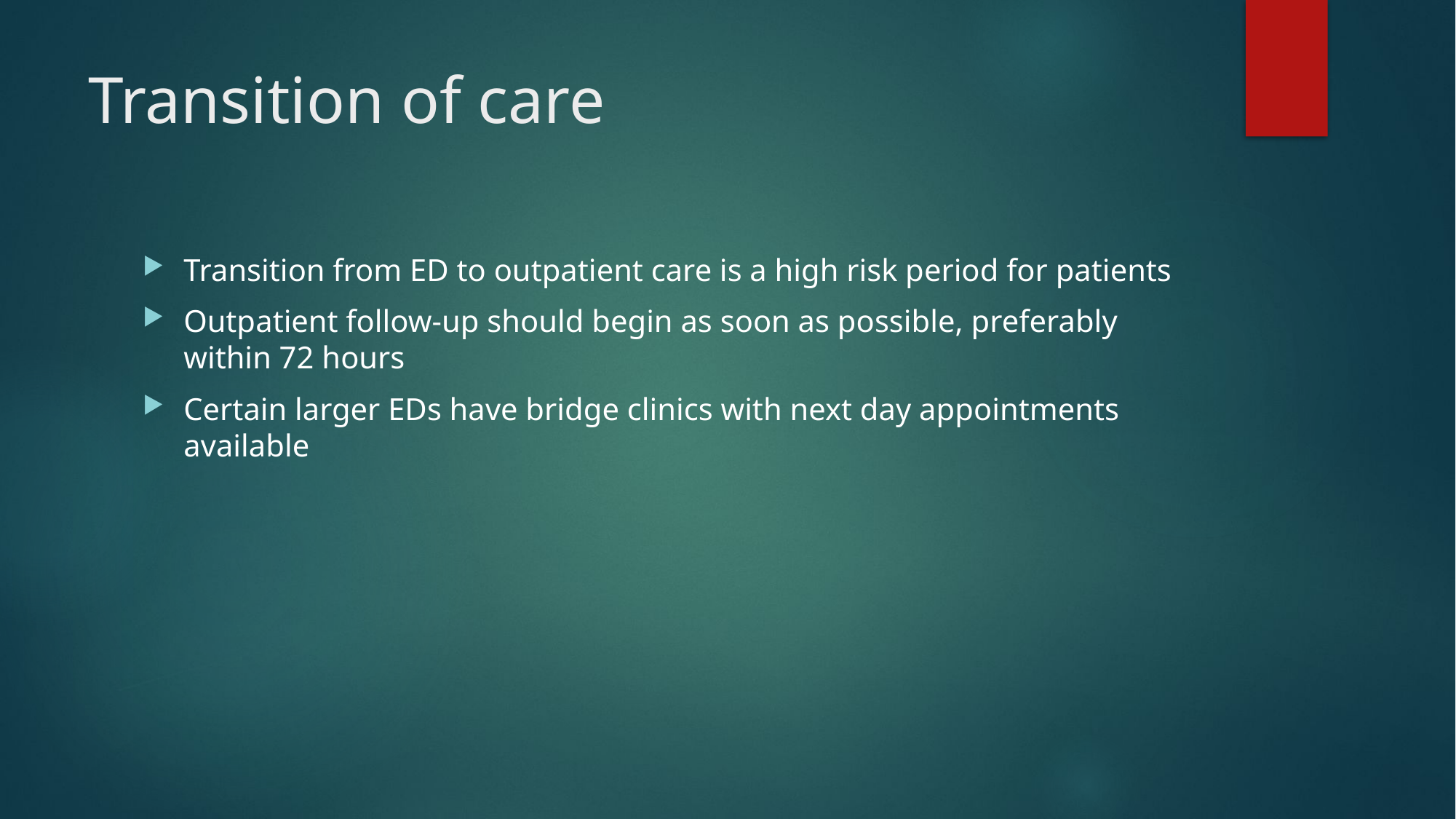

# Transition of care
Transition from ED to outpatient care is a high risk period for patients
Outpatient follow-up should begin as soon as possible, preferably within 72 hours
Certain larger EDs have bridge clinics with next day appointments available

## Slide 28
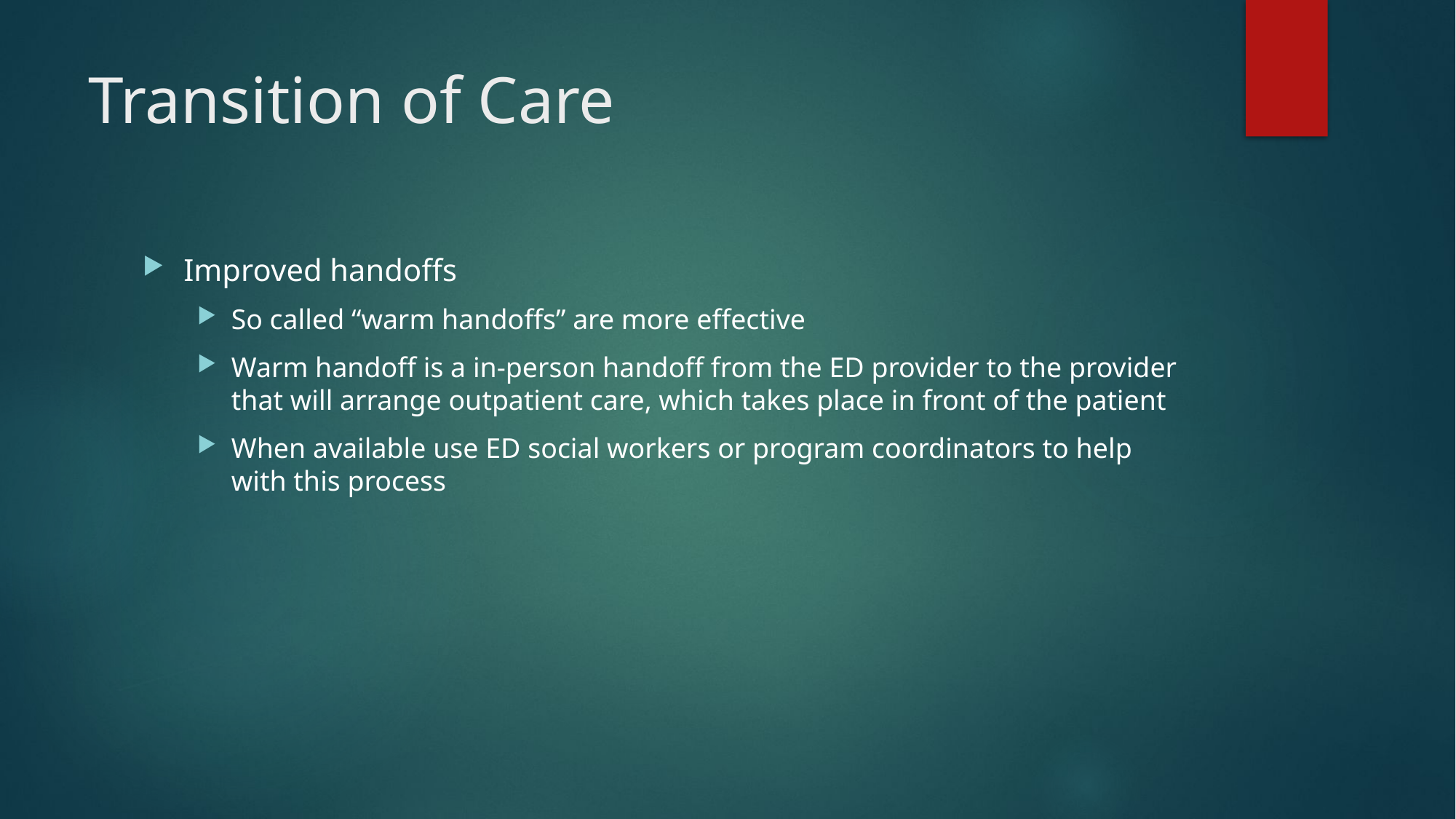

# Transition of Care
Improved handoffs
So called “warm handoffs” are more effective
Warm handoff is a in-person handoff from the ED provider to the provider that will arrange outpatient care, which takes place in front of the patient
When available use ED social workers or program coordinators to help with this process

## Slide 29
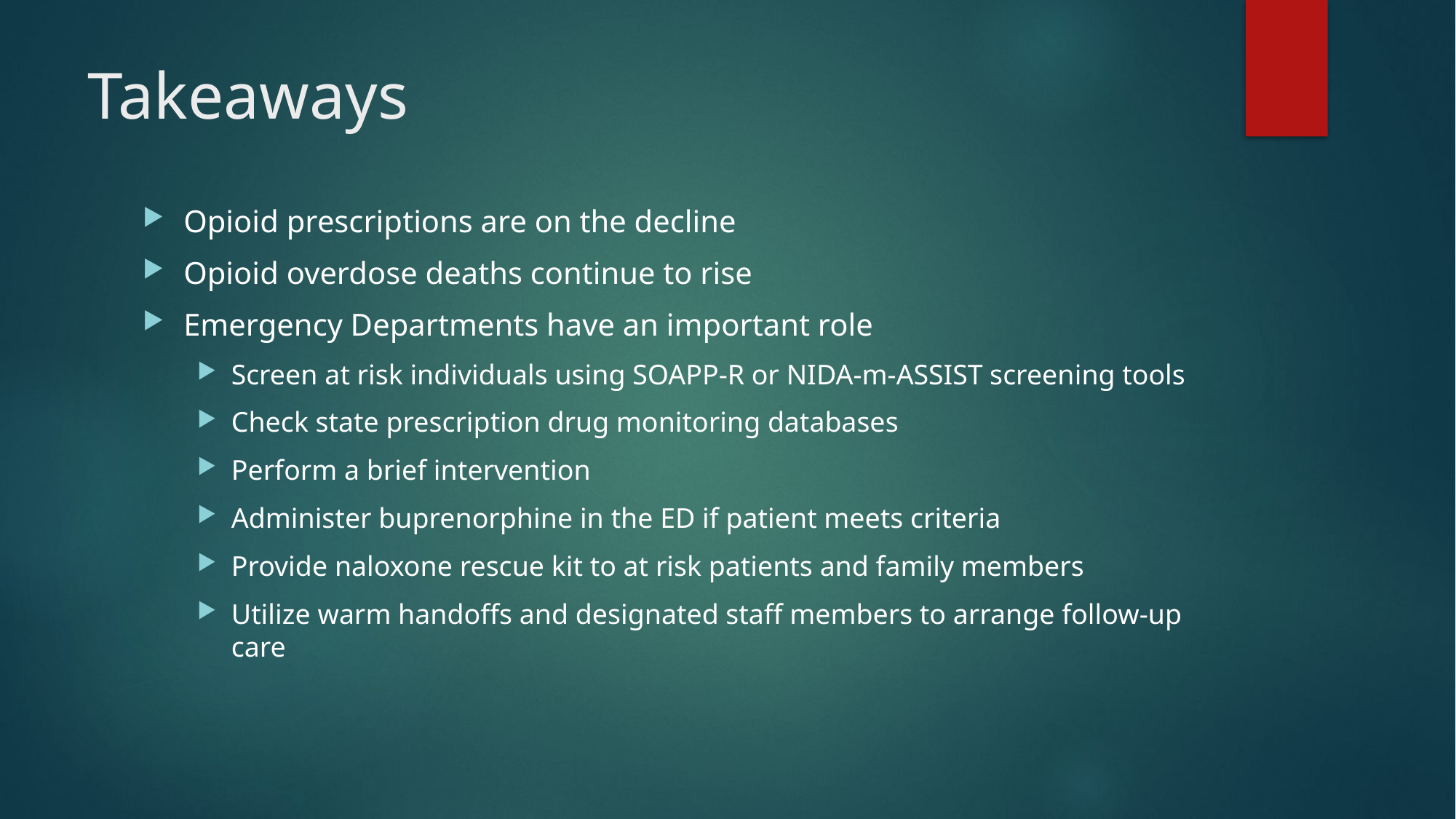

# Takeaways
Opioid prescriptions are on the decline
Opioid overdose deaths continue to rise
Emergency Departments have an important role
Screen at risk individuals using SOAPP-R or NIDA-m-ASSIST screening tools
Check state prescription drug monitoring databases
Perform a brief intervention
Administer buprenorphine in the ED if patient meets criteria
Provide naloxone rescue kit to at risk patients and family members
Utilize warm handoffs and designated staff members to arrange follow-up care

## Slide 30
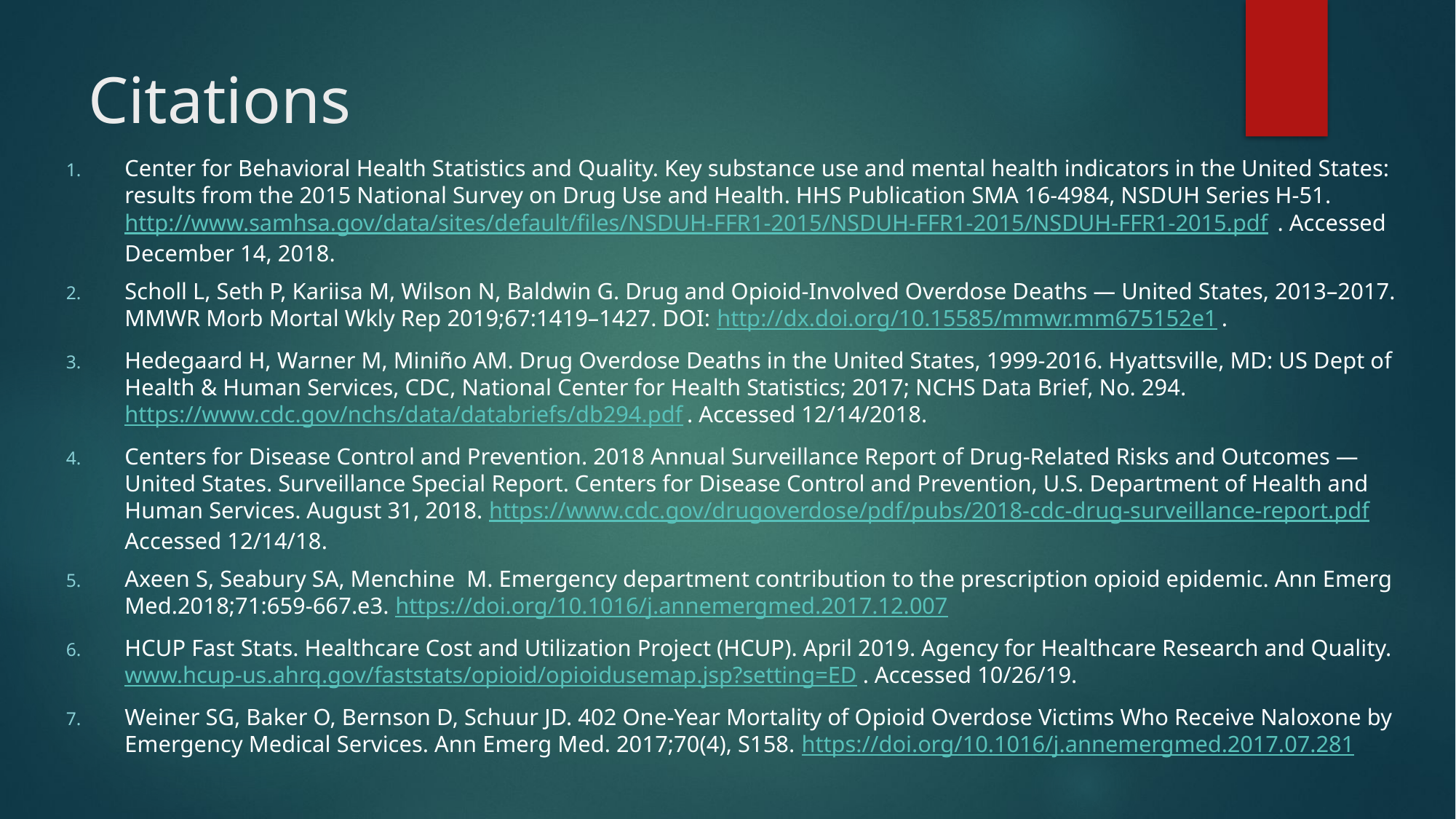

# Citations
Center for Behavioral Health Statistics and Quality. Key substance use and mental health indicators in the United States: results from the 2015 National Survey on Drug Use and Health. HHS Publication SMA 16-4984, NSDUH Series H-51. http://www.samhsa.gov/data/sites/default/files/NSDUH-FFR1-2015/NSDUH-FFR1-2015/NSDUH-FFR1-2015.pdf. Accessed December 14, 2018.
Scholl L, Seth P, Kariisa M, Wilson N, Baldwin G. Drug and Opioid-Involved Overdose Deaths — United States, 2013–2017. MMWR Morb Mortal Wkly Rep 2019;67:1419–1427. DOI: http://dx.doi.org/10.15585/mmwr.mm675152e1.
Hedegaard H, Warner M, Miniño AM. Drug Overdose Deaths in the United States, 1999-2016. Hyattsville, MD: US Dept of Health & Human Services, CDC, National Center for Health Statistics; 2017; NCHS Data Brief, No. 294. https://www.cdc.gov/nchs/data/databriefs/db294.pdf. Accessed 12/14/2018.
Centers for Disease Control and Prevention. 2018 Annual Surveillance Report of Drug-Related Risks and Outcomes — United States. Surveillance Special Report. Centers for Disease Control and Prevention, U.S. Department of Health and Human Services. August 31, 2018. https://www.cdc.gov/drugoverdose/pdf/pubs/2018-cdc-drug-surveillance-report.pdf Accessed 12/14/18.
Axeen S, Seabury SA, Menchine M. Emergency department contribution to the prescription opioid epidemic. Ann Emerg Med.2018;71:659-667.e3. https://doi.org/10.1016/j.annemergmed.2017.12.007
HCUP Fast Stats. Healthcare Cost and Utilization Project (HCUP). April 2019. Agency for Healthcare Research and Quality. www.hcup-us.ahrq.gov/faststats/opioid/opioidusemap.jsp?setting=ED. Accessed 10/26/19.
Weiner SG, Baker O, Bernson D, Schuur JD. 402 One-Year Mortality of Opioid Overdose Victims Who Receive Naloxone by Emergency Medical Services. Ann Emerg Med. 2017;70(4), S158. https://doi.org/10.1016/j.annemergmed.2017.07.281

## Slide 31
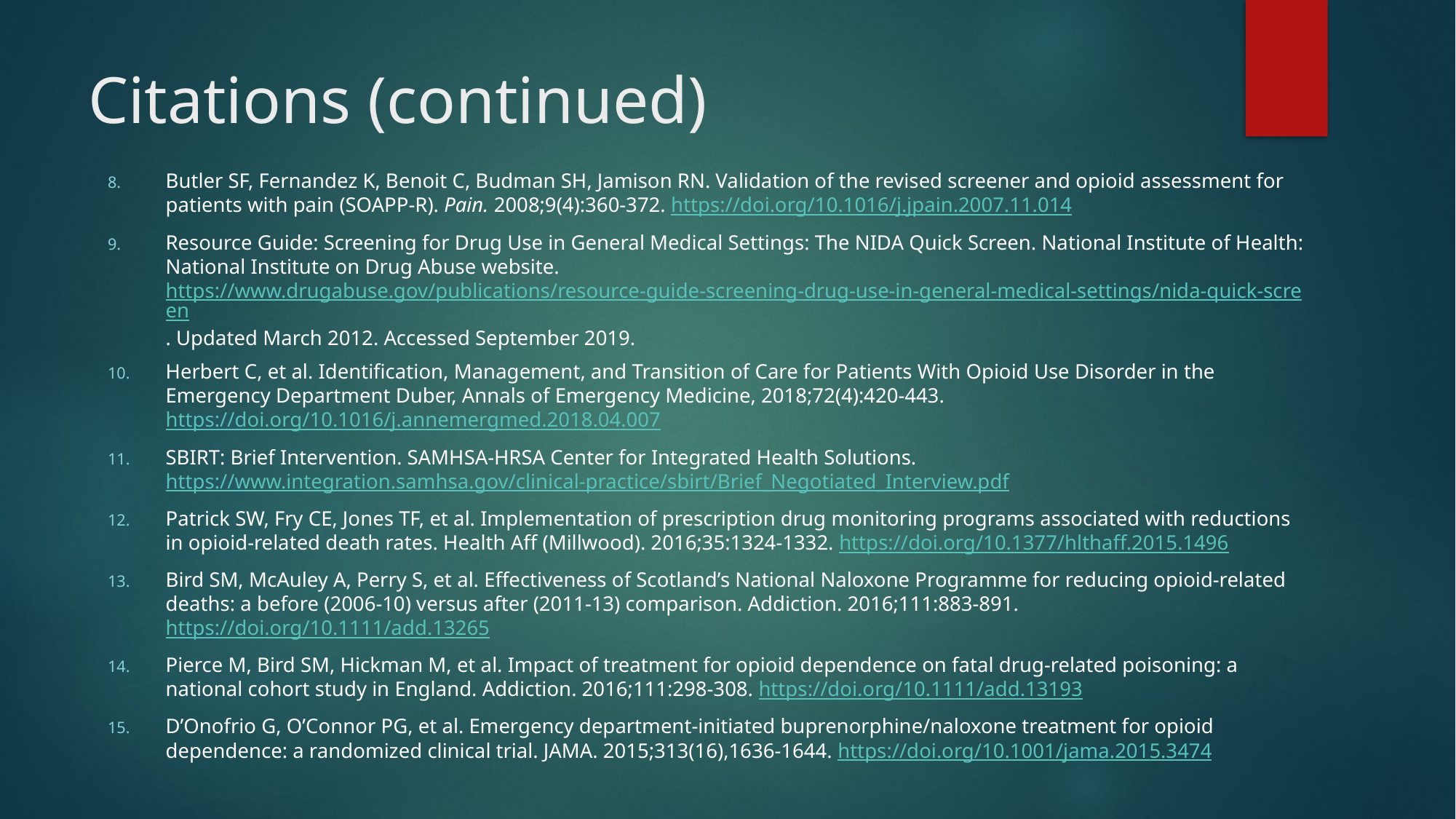

# Citations (continued)
Butler SF, Fernandez K, Benoit C, Budman SH, Jamison RN. Validation of the revised screener and opioid assessment for patients with pain (SOAPP-R). Pain. 2008;9(4):360-372. https://doi.org/10.1016/j.jpain.2007.11.014
Resource Guide: Screening for Drug Use in General Medical Settings: The NIDA Quick Screen. National Institute of Health: National Institute on Drug Abuse website. https://www.drugabuse.gov/publications/resource-guide-screening-drug-use-in-general-medical-settings/nida-quick-screen. Updated March 2012. Accessed September 2019.
Herbert C, et al. Identification, Management, and Transition of Care for Patients With Opioid Use Disorder in the Emergency Department Duber, Annals of Emergency Medicine, 2018;72(4):420-443. https://doi.org/10.1016/j.annemergmed.2018.04.007
SBIRT: Brief Intervention. SAMHSA-HRSA Center for Integrated Health Solutions. https://www.integration.samhsa.gov/clinical-practice/sbirt/Brief_Negotiated_Interview.pdf
Patrick SW, Fry CE, Jones TF, et al. Implementation of prescription drug monitoring programs associated with reductions in opioid-related death rates. Health Aff (Millwood). 2016;35:1324-1332. https://doi.org/10.1377/hlthaff.2015.1496
Bird SM, McAuley A, Perry S, et al. Effectiveness of Scotland’s National Naloxone Programme for reducing opioid-related deaths: a before (2006-10) versus after (2011-13) comparison. Addiction. 2016;111:883-891. https://doi.org/10.1111/add.13265
Pierce M, Bird SM, Hickman M, et al. Impact of treatment for opioid dependence on fatal drug-related poisoning: a national cohort study in England. Addiction. 2016;111:298-308. https://doi.org/10.1111/add.13193
D’Onofrio G, O’Connor PG, et al. Emergency department-initiated buprenorphine/naloxone treatment for opioid dependence: a randomized clinical trial. JAMA. 2015;313(16),1636-1644. https://doi.org/10.1001/jama.2015.3474
