# Supplementary material for: Identification and Treatment of Opioid Withdrawal and Opioid Use Disorder in the Emergency Department
Source: MedEdPORTAL. 2020 May 15;16:10899. doi: 10.15766/mep_2374-8265.10899 (PMC7331957; doi:10.15766/mep_2374-8265.10899)
Supplement: Supplementary file 1 — OUD in the ED Introduction.pptxOUD Case - Facilitator.docxOUD Case - Trainee.docxTest Questions.docxTest Questions Answer Key.docx [file mep_2374-8265.10899-s001.zip › B. OUD Case - Facilitator.docx]

Emergency Department Triage Note:

34 yo M

Abdominal cramping, nausea, vomiting, diarrhea, and chills x 2 days

BP 122/78, HR 123, RR 18, 98% on room air, Temperature 37.1 C

Your attending asks you to go see the patient and complete a history and physical exam. What components of the History of Present Illness would you like to ask, and what other parts of the history are important?

The HPI should address: the onset of symptoms, duration of symptoms (although this is in the triage summary), characteristics of the symptoms, alleviating and aggravating factors for the symptoms, timing and severity

Other important parts of the history are the past medical history, past surgical history, medications, allergies, family history, and social history. It is essential for the students in this case to think about the drug use section of the social history. If the group is not hitting on this key point, prompt them to consider asking about alcohol, smoking, and recreational drug use.

During your interview the patient reports 2 days of constant, gradually worsening chills, sweats, nausea, vomiting, diarrhea, and abdominal pain. The abdominal pain is crampy, diffuse, non-radiating, 10/10 on the pain scale, and unchanged with eating or movement. The patient has had 6 episodes of emesis per day, which have been watery without evidence of bile or blood. He also reports 20 episodes of watery, non-bloody, non-melanotic diarrhea yesterday. The amount of diarrhea has gradually been increasing. Nothing has really made any of the symptoms better. Trying to eat seems to make the nausea, but not the abdominal pain, worse.

Past Medical History: asthma

Past Surgical History: tonsillectomy at age 5

Allergies: none

Medications: none

Family History: Father died a motor vehicle crash 2 years ago, Mother has diabetes and hypertension

Social History: Lives with three roommates in an apartment with city water. Works as a waiter at a local restaurant. He has smoked 1 pack per day for the past 12 years. When ask about drug use, the patient becomes guarded.

What strategies can you use to obtain sensitive information, like recreational drug use, from a patient?

- Normalizing: using universal statements to normalize the problem and/or anxiety about discussing the issue
  - Example: “Many people find it difficult to talk about substance misuse, particularly for things that may be illegal.” Or “We ask everyone about this because it is important to insure we give patients the best care”
- Transparency: Explain why you asking the information and why it is important for their medical care
  - Example: “It is important that we know about what substances you have been using because it could be related to your current complaint and will allow us to more accurately diagnose and appropriately treat the problem.
- Asking permission: Ask the patient if you can discuss a sensitive topic
- Give the option of not answering the question
- Confidentiality: legally must be maintained per the Healthcare Information Portability and Accountability Act (HIPPA), except in some cases such as child and elder abuse and neglect
- Consider asking yes/no questions, or questions with limited choices (never, sometimes, always, almost always) to reduce anxiety

What screening tools have been validated in the Emergency Department setting for screening for potentially dangerous opioid use?

- Revised Screener and Opioid Assessment for Patients with Pain (SOAPP-R)
- NIDA quick screen

After your discussion with the patient, he reports has been injecting heroin 1-2 times per day or taking whatever opiate is available by mouth. You thank the patient for sharing that information and move on to your physical exam.

- BP 122/78, HR 123, RR 18, 98% on room air, Temperature 37.1 C
- General: an uncomfortable appearing male, slightly diaphoretic and holding his abdomen
- Head: Normocephalic and atraumatic
- Ears, nose, throat: TMs clear bilaterally, oropharynx clear without exudates or swelling, full range of motion of the neck, no cervical adenopathy
- Eyes: Pupils 6mm and equal bilaterally, reactive to light. Conjunctive injected
- Lungs: mostly clear throughout with a few scattered wheezes
- Cardiovascular: strong and equal pulses in all extremities. Heart with regular rhythm, tachycardic rate. No murmurs, rubs, or gallops
- Abdomen: hyperactive bowel sounds throughout, mild and diffuse tenderness. No rebound or guarding
- Genitourinary: deferred
- Skin: erythema and signs of prior injection in the bilateral antecubital fossae and forearms. No abscess or cellulitis. No splinter hemorrhages. Mild piloerection diffusely
- Neurologic: CN 2-12 intact, 5/5 strength and sensation intact in all 4 extremities

What is your differential?

- Viral gastroenteritis
- Clostridium difficile
- Colitis
- Pancreatitis
- Opioid withdrawal

What work up would you consider?

- CBC
- CMP
- Lipase
- Lactate
- UA
- CT of the abdomen and pelvis
- C diff testing
- Troponin
- EKG

What medications or interventions could you start at this point?

- Intravenous fluids (normal saline or lactated ringers)
- Anti-emetics: ondansetron, promethazine, metoclopramide
- Anti-diarrheal: Loperamide (avoid if you think it is c. diff)
- Pain management: always consider non-opiate pain control strategies first
- Specifically, for opioid withdrawal: clonidine, buprenorphine

All lab work and imaging is unremarkable. The history and time course are consistent with opiate withdrawal, and the patient states this feels like prior withdrawal episodes. The patient has already screened “positive” through the NIDA quick screening, using opiates multiple times per day. What is the next step in SBIRT? Give examples of how you would have that conversion.

1. Raise the subject: The first step is establishing rapport and raising the subject with the patient. Some patients will be more open to discussion of sensitive topics than others. Establishing rapport is an important part of SBIRT and can be achieved by attempting to understand the patient’s circumstances and avoiding a judgmental stance. Examples of how to raise the subject include:

- Would you mind taking a few minutes to talk with me about your opioid use?
- May I discuss your opioid use with you?

Following the patient’s agreement to discuss his or her drug and/or alcohol use, you can discuss the frequency and quantity of the patient’s use, pros and cons of the patients use, and re-state the findings for the patient. Should the patient decline the discussion, respect the patient’s decision and let them know our availability should that decision change. For example, “I understand you aren’t ready to discuss this today, but we are available should you change your mind”.

1. Provide Feedback: In this step the provider is summarizing the discussion that has already occurred and providing the patient with information about health risks associated with the quantity or type of substance use. Steps to this process include:
   1. Reviewing the patient’s screen with them and/or reviewing their substance use patterns
   2. Make a connection to the patient’s acute health crisis that caused their presentation to the emergency department. Examples of ways to ask this include:

- What connection do you see between your drug use and this ED visit?
- Do you think this ED visit could be related to your substance use?

If the patient sees a connection between their substance use and the ED visit, reiterate it. If the patient does not see a connection, make one for them using facts.

1. Enhance motivation: The focus of this step is to increase the patient’s motivation to reduce or stop their substance use or enter treatment if appropriate.
   1. Readiness to change: On a scale of 1 to 10, how ready are you to change your drug use?
      1. If the patient picks a low number, ask if the patient has ever done something they regretted as a result of substance use, and again discuss pros and cons
      2. If the patient picks a high number, ask the patient the reasons they are thinking about changing
      3. For patients not ready for change, do not shame or confront and avoid arguments
   2. Develop discrepancy:
      1. Not ready to change: Acknowledge that not being ready to change is ok, attempt to motivate
         1. What would make your drug use a problem for you?
         2. How important would it be to prevent that thing from happening?
         3. Discuss the pros and the cons of continued use
      2. Ready to change:
         1. Why do you want to change this aspect?
         2. What barriers to do you have to change?
         3. What benefits are there to change?
   3. Negotiate and advise: In this step, we attempt to get the patient to reflect on the conversation. This can be done by asking “What are your thoughts on all of this?” or “How does this sound to you?”.
      1. Summarize: From what I’ve heard you say, you are prepared to…
      2. What is the next step? Discuss options available to the patient:
         1. Outpatient referrals for treatment program
         2. ED buprenorphine if available with appropriate prescription and/or follow up^[[1]](#footnote-1)^

After completing your Brief Intervention, the patient has indicated that we would like treatment for his Opioid Use Disorder. You are working in an Emergency Department that has an ED buprenorphine program. What things would exclude him from being able to get buprenorphine in the ED and what additional information do you need to know?

Exclusion: COWS <8, caution in patients with significant benzodiazepine use

Need to know: last opiate he took, the time, and the dose (if possible)

The COWS is calculated at 12. The urine drug screen in negative for benzodiazepines and the patient denies any significant alcohol or benzodiazepine use. The patient reports he last used heroin 14 hours ago. Given this information, the patient’s commitment to treating his opiate use disorder, and that he understands the treatment follow up and plan, the patient is given his first dose of buprenorphine in the Emergency Department. You nurse comes and asks “Hey I thought you couldn’t give that medication without a special license” and requests further information regarding the regulations about buprenorphine administration and prescription. What do you tell him?

Starting and maintaining patients on buprenorphine usually requires a Drug Addiction Treatment Act of 2000 (DATA 2000) X-waiver, which is obtained after an 8-hour training program for physicians. However, the “72-hour rule” or “3 day rule” (Title 21, Code of Federal Regulations, Part 1306.07) allows physicians to administer (give the medication to the patient in front of them and watch them take it) buprenorphine for up to 72 hours to treat opiate withdrawal and addiction, without having completed the additional training

Physicians CANNOT write a prescription for buprenorphine to treat opioid addiction, NOR can a physician dispense (give the patient a medication to go home with) without completing the DATA 2000 training.

Thirty minutes after the buprenorphine was given, the patient’s COWS decreased from 12 to 2. The patient followed up as scheduled the next day, and remains in treatment when you call to check on him at 30 days after you saw him in the ED.

1. D’Onofrio G, Pantalon MV, Degutis LC, Fiellin DA, O’Connor PG. Project ED Health, NIAAA. [www.medicine,yale.edu/sbirt/images/sbirt_script_7jul09_tcm508-100698.pdf](http://www.medicine,yale.edu/sbirt/images/sbirt_script_7jul09_tcm508-100698.pdf). Accessed 10/27/19. [↑](#footnote-ref-1)
