# Supplementary material for: Identification and Treatment of Opioid Withdrawal and Opioid Use Disorder in the Emergency Department
Source: MedEdPORTAL. 2020 May 15;16:10899. doi: 10.15766/mep_2374-8265.10899 (PMC7331957; doi:10.15766/mep_2374-8265.10899)
Supplement: Supplementary file 1 — OUD in the ED Introduction.pptxOUD Case - Facilitator.docxOUD Case - Trainee.docxTest Questions.docxTest Questions Answer Key.docx [file mep_2374-8265.10899-s001.zip › C. OUD Case - Trainee.docx]

Emergency Department Triage Note:

34 yo M

Abdominal cramping, nausea, vomiting, diarrhea, and chills x 2 days

BP 122/78, HR 123, RR 18, 98% on room air, Temperature 37.1 C

Your attending asks you to go see the patient and complete a history and physical exam. What components of the History of Present Illness would you like to ask, and what other parts of the history are important?

During your interview the patient reports 2 days of constant, gradually worsening chills, sweats, nausea, vomiting, diarrhea, and abdominal pain. The abdominal pain is crampy, diffuse, non-radiating, 10/10 on the pain scale, and unchanged with eating or movement. The patient has had 6 episodes of emesis per day, which have been watery without evidence of bile or blood. He also reports 20 episodes of watery, non-blood, non-melanotic diarrhea yesterday. The amount of diarrhea has gradually been increasing. Nothing has really made any of the symptoms better. Trying to eat seems to make the nausea, but not the abdominal pain, worse.

Past Medical History: asthma

Past Surgical History: tonsillectomy at age 5

Allergies: none

Medications: none

Family History: Father died a motor vehicle crash 2 years ago, Mother has diabetes and hypertension

Social History: Lives with three roommates in an apartment with city water. Works as a waiter at a local restaurant. He has smoked 1 pack per day for the past 12 years. When ask about drug use, the patient becomes guarded.

What strategies can you use to obtain sensitive information, like recreational drug use, from a patient?

What screening tools have been validated in the Emergency Department setting for screening for potentially dangerous opioid use?

After your discussion with the patient, he reports has been injecting heroin 1-2 times per day or taking whatever opiate is available by mouth. You thank the patient for sharing that information and move on to your physical exam.

- BP 122/78, HR 123, RR 18, 98% on room air, Temperature 37.1 C
- General: an uncomfortable appearing male, slightly diaphoretic and holding his abdomen
- Head: Normocephalic and atraumatic
- Ears, nose, throat: TMs clear bilaterally, oropharynx clear without exudates or swelling, full range of motion of the neck, no cervical adenopathy
- Eyes: Pupils 6mm and equal bilaterally, reactive to light. Conjunctive injected
- Lungs: mostly clear throughout with a few scattered wheezes
- Cardiovascular: strong and equal pulses in all extremities. Heart with regular rhythm, tachycardic rate. No murmurs, rubs, or gallops
- Abdomen: hyperactive bowel sounds throughout, mild and diffuse tenderness. No rebound or guarding
- Genitourinary: deferred
- Skin: erythema and sings of prior injection in the bilateral antecubital fossae and forearms. No abscess or cellulitis. No splinter hemorrhages. Mild piloerection diffusely
- Neurologic: CN 2-12 intact, 5/5 strength and sensation intact in all 4 extremities

What is your differential?

What work up would you consider?

What medications or interventions could you start at this point?

All lab work and imaging is unremarkable. The history and time course are consistent with opiate withdrawal, and the patient states this feels like prior withdrawal episodes. The patient has already screened “positive” through the NIDA quick screening, using opiates multiple times per day. What is the next step in SBIRT? Give examples of how you would have that conversion.

After completing your Brief Intervention, the patient has indicated that we would like treatment for his Opioid Use Disorder. You are working in an Emergency Department that has an ED buprenorphine program. What things would exclude him from being able to get buprenorphine in the ED and what additional information do you need to know?

The COWS is calculated at 12. The urine drug screen in negative for benzodiazepines and the patient denies any significant alcohol or benzodiazepine use. The patient reports he last used heroin 14 hours ago. Given this information, the patient’s commitment to treating his opiate use disorder, and that he understands the treatment follow up and plan, the patient is given his first dose of buprenorphine in the Emergency Department. You nurse comes and asks “Hey I thought you couldn’t give that medication without a special license” and requests further information regarding the regulations about buprenorphine administration and prescription. What do you tell him?

Thirty minutes after the buprenorphine was given, the patient’s COWS decreased from 12 to 2. The patient followed up as scheduled the next day, and remains in treatment when you call to check on him at 30 days after you saw him in the ED.
