# Supplementary material for: Identification and Treatment of Opioid Withdrawal and Opioid Use Disorder in the Emergency Department
Source: MedEdPORTAL. 2020 May 15;16:10899. doi: 10.15766/mep_2374-8265.10899 (PMC7331957; doi:10.15766/mep_2374-8265.10899)
Supplement: Supplementary file 1 — OUD in the ED Introduction.pptxOUD Case - Facilitator.docxOUD Case - Trainee.docxTest Questions.docxTest Questions Answer Key.docx [file mep_2374-8265.10899-s001.zip › E. Test Questions Answer Key.docx]

Name________________________

COM Class of _________________

Opioid Use Disorder (OUD) in the Emergency Department

1. Emergency departments are responsible for approximately what percentage of opioids prescribed?

**A) 5%**

B) 10%

C) 20%

D) 40%

1. Approximately how many people died from drug overdose deaths in 2017?

A) 30,000

B) 50,000

**C) 70,000**

D) 100,000

1. Opioid overdoses killed more people in the US than motor vehicle crashes in 2017

**A) True**

B) False

1. Opioid prescription rates over the past 10 years have

A) increased

**B) decreased**

C) stayed constant

1. Which drug class is responsible for the most overdose deaths?

A) Prescription opioids (natural and semi-synthetic)

B) Heroin

**C) Synthetic opioids other than methadone (ie. fentanyl)**

D) Cocaine

1. Who does ACEP recommend screening for OUD in the emergency setting?

A) Everyone

B) Men between the ages of 18-45

C) Men and women between the ages of 18-45

**D) At risk individuals**

1. Which OUD screening tool has been validated in an emergency department setting?

**A) SOAPP-R (Revised screener and opioid assessment for patients with pain)**

B) ABC (Addiction behaviors checklist)

C) ORT (Opioid risk tool)

D) COMM (Current opioid misuse measure)

1. Which medication assisted therapy does ACEP currently recommend in the emergency setting?

A) Methadone

**B) Buprenorphine**

C) Naltrexone

D) Hydromorphone

1. Studies have shown which of the following to be the most effective treatment for OUD in the ED

A) Screening and referral to treatment

B) Screening, brief intervention, and referral to treatment

**C) Screening, brief intervention, ED initiation of medication assisted therapy, and referral to treatment**

1. Which of the following medications can induce acute opioid withdrawal?

A) methadone

**B) buprenorphine**

C) fentanyl

D) hydromorphone

1. What are important factors to consider prior to giving buprenorphine in the ED?
2. the last opiate taken
3. the time the last opiate was taken
4. the Clinical Opiate Withdrawal Scale (COWS)
5. **All of the above**
6. Which of the follow is true of the Clinical Opiate Withdrawal Scale (COWS)?

**A) Scores range from 0-36**

B) A score of 0 indicates maximum withdrawal symptoms

C) It is based on symptoms in 6 different categories

D) It shouldn’t be used as a decision aid for initiating medication assisted therapy

1. In which of the following scenarios should you administer buprenorphine?

A) Patient who used heroin 5 hours ago and COWS 6

B) Patient who used methadone 2 days ago and COWS 2
**C) Patient who used OxyContin® 24 hours ago and COWS 8**

1. Providers without a DEA X-license may

A) Prescribe methadone or buprenorphine indefinitely

B) Dispense 72 hours’ worth of methadone or buprenorphine from the ED

**C) Administer methadone or buprenorphine in the ED**

D) Not administer methadone or buprenorphine at all
