# Supplementary material for: Identification and Treatment of Opioid Withdrawal and Opioid Use Disorder in the Emergency Department
Source: MedEdPORTAL. 2020 May 15;16:10899. doi: 10.15766/mep_2374-8265.10899 (PMC7331957; doi:10.15766/mep_2374-8265.10899)
Supplement: Supplementary file 1 — OUD in the ED Introduction.pptxOUD Case - Facilitator.docxOUD Case - Trainee.docxTest Questions.docxTest Questions Answer Key.docx [file mep_2374-8265.10899-s001.zip › _Educational_Summary_Report_10899.pdf]

MedEdPORTAL® The AAMC Journal of Teaching and Learning Resources

Original Publication Open Access

Identification and Treatment of Opioid Withdrawal and Opioid Use Disorder in the Emergency Department

Lindsey Jennings, MD, MPH*, Tessa Warner, MD, MPH, Bastien Bacro-Duverger, MD

*Corresponding author: jennil@musc.edu

Abstract

Introduction: Opioid addiction and misuse constitute a public health crisis in the United States. Recent research supports buprenorphine induction in the emergency department (ED) setting for patients with opioid use disorder (OUD). However, education regarding buprenorphine induction for emergency medicine (EM) physicians, residents, and students is still limited. **Methods:** We created a 1-hour introductory workshop for the identification and treatment of opioid withdrawal and OUD in the ED for medical students going into EM and for EM interns. The workshop consisted to two distinct curricular sections: (1) a didactic session providing an overview of the basic knowledge and skills to identify and treat patients with OUD in the ED and (2) a case-based session in which students worked through the identification of opioid withdrawal; discussion of opioid use with the patient, going through the steps of SBIRT (screening, brief intervention, and referral to treatment); and considerations when starting buprenorphine. The workshop was evaluated using a pre- and posttest examining medical knowledge around buprenorphine use in the ED. **Results:** A total of 48 students and interns participated in the curriculum. The students showed a significant improvement in medical knowledge between the pre- and posttests, with an average 45% higher score on the posttest ($p < .001$). **Discussion:** Our workshop resulted in a short-term improvement in medical students' and interns' knowledge of the identification and treatment of patients with OUD in the ED.

Keywords
Opioids, Opioid Use Disorder, SBIRT, Emergency Medicine, Case-Based Learning

Educational Objectives

By the completion of the workshop, participants should be able to:

1. Explain the current state of the opioid epidemic in the US.
2. List which drug class is responsible for the most overdose deaths.
3. Describe who to screen for opioid use disorder and how to screen them in an emergency department (ED) setting.
4. Summarize the use of medication-assisted treatment from the ED.
5. List important considerations prior to giving buprenorphine in the ED.
6. Explain what physician is allowed to do with a Drug Addiction Treatment Act X-license.
7. Apply acquired knowledge to a practice case.

Citation:
Jennings L, Warner T, Bacro-Duverger B. Identification and treatment of opioid withdrawal and opioid use disorder in the emergency department. *MedEdPORTAL*. 2020;16:10899.
https://doi.org/10.15766/mep_2374-8265.10899

Introduction

Opioid misuse is a public health crisis in the United States. In 2015, 3.8 million people in the US misused pain medications, and an additional 329,000 used heroin.[1,2] In 2016, there were over 60,000 drug overdose deaths, a 200% increase since 2000.[3,4] Within the US health care system, emergency departments (EDs) are at the frontline of the opioid epidemic. ED visits related to opioid use tripled between 2008 and 2016.[5] Not only are EDs seeing more patients with opioid use disorder (OUD), they are also treating patients with very severe disease. One study found that one out of every 10 patients reversed with naloxone by emergency medical services died within 12 months of reversal.[6]

Currently, there is a significant gap between the number of patients who need treatment for OUD and the number in treatment.[7] In response to this treatment gap, research and quality improvement efforts have focused on connecting patients to treatment from the ED setting. In 2015, a practice-changing study demonstrated that ED-based buprenorphine induction, brief intervention, and referral to treatment was significantly more effective at retaining patients in treatment compared to the standard of care, which is outpatient referral.[8] In response, many

Copyright © 2020 Jennings et al. This is an open-access publication distributed under the terms of the Creative Commons Attribution license.

EDs expanded their role in the treatment of OUD by screening, initiating buprenorphine, and arranging expeditious outpatient follow-up. While these efforts have significantly improved the quality of care provided to patients in the ED setting, this process is still very new. As a result, education about ED-initiated treatments for patients with OUD is needed. Medical students, residents, and attending physicians require further education about the benefits of this treatment and how to safely implement these strategies in their daily practice.

At our institution, we started an ED-initiated buprenorphine program in December of 2017 and have been actively developing educational curricula for our attending physicians and residents. The program has been a significant area of interest for medical students heading into emergency medicine (EM). In response, we developed a 45-minute didactic session, followed by a case-based exercise aimed at educating medical students and residents about ED-based treatments for opioid abuse.

Several curricular and educational activities teaching medical students and residents about opioid use have been published. However, very few curricula are focused on addressing opioid addiction in the ED setting, and none of the curricula published to date include detailed information about starting patients on buprenorphine.[9,10]

Methods

We created a 1-hour introductory workshop for the identification and treatment of opioid withdrawal and OUD in the ED setting for medical students and EM interns. The curriculum was delivered to participants in five different sessions. Of those sessions, one was for medical students (years 1-4) at an EM interest group meeting, three were didactic sessions for the fourth-year EM-bound students on their EM subinternship, and the last was delivered to EM interns during their orientation month in July. All sessions contained eight to 11 participants. The workshop was developed and implemented by a fourth-year medical student, a senior-year EM resident, and an EM faculty member. Materials required for the workshop included paper, pens, a projector, and a computer.

The workshop consisted to two distinct curricular sections. The first was a 30-minute didactic session providing an overview of the basic knowledge and skills needed to identify and treat patients with OUD in the ED. Specifically, the presentation (Appendix A) reviewed the opioid epidemic, some initial failed attempts to address the problem, and the role of EM in addressing the opioid epidemic. The didactic session also gave an overview of the screening, brief intervention, and referral to treatment (SBIRT) model by discussing screening mechanisms tested in EDs,[11,12] techniques to engage patients in discussing their substance misuse, and the basic steps of a brief negotiated interview. A significant portion of the didactic session was dedicated to medication-assisted treatment. Focusing on buprenorphine, we reviewed the biochemical mechanism, the evidence for its use in the ED, and considerations for initiating use, including the Clinical Opiate Withdraw Scale (COWS).[13] The last portion of the didactic session covered the transition of care of the patient to the outpatient setting and regulatory issues surrounding buprenorphine and the Drug Addiction Treatment Act of 2000 X-waiver.

Following a 5-minute break, we began the second curricular section, which was a case-based session. The facilitators helped guide the case to completion using the prompts detailed in the facilitator copy of the case (Appendix B). The learners each received a copy of the case handout (Appendix C) to work through the identification of opioid withdrawal, discuss opioid use with the patient, practice the SBIRT method, and discuss considerations for starting buprenorphine. The case-based session was completed in groups of two to three students or residents. In the session for students at the EM interest group meeting, each group was led by one of the three facilitators (fourth-year medical student, EM resident, or EM faculty member). At all the other sessions, one facilitator (EM attending physician) oversaw all groups during the case. The case took approximately 20 minutes for all groups to complete.

To evaluate the workshop, the same test (Appendix D) was used pre- and postworkshop to determine whether the educational objectives had been attained and if the trainees had gained knowledge from the session. The pre- and posttests were developed by the EM attending to assess major learning points from the workshop. No pilot testing was used prior to implementation. The pre- and posttests were scored per the answer key (Appendix E). In both the pre- and posttest, participants were asked 14 knowledge questions about the principles of identification and management of buprenorphine induction.

Results

The curriculum was delivered to 38 medical students, predominately from year 4, and 10 EM interns, for a total of 48 participants. All participants completed the pre- and postworkshop tests. The tests were taken anonymously, and pre- and posttests were matched.

Copyright © 2020 Jennings et al. This is an open-access publication distributed under the terms of the Creative Commons Attribution license.

In the preworkshop test, scores ranged from 7% to 71% correctly answered questions, with an average of 44%. The postworkshop test scores ranged from 71% to 100% correctly answered questions, with an average of 89%. The postworkshop test score average was 45 percentage points higher than the preworkshop test score average. Postworkshop test scores improved 29 to 79 percentage points from preworkshop test scores. Pre- and postworkshop test scores were compared using a paired, two-tailed *t* test, resulting in a p value of $<.0001$, which was considered to be statistically significant.

Discussion

We developed a workshop to serve as an introduction to the identification and treatment of opioid withdrawal and addiction in the ED setting. The workshop showed a significant improvement in student and resident knowledge. The workshop begins to address the need for educational curricula regarding opioid withdrawal and OUD in the ED setting. As we educate more students, residents, and physicians about safely starting buprenorphine in EDs, we can better address the needs of our patients.

Strengths of the curriculum include its succinctness, its inclusion of buprenorphine induction, its interactive case to apply and solidify the knowledge gained, and its focus on engaging patients in discussing opioid misuse through patient-centered communication strategies. Given the lack of need for significant simulation or expensive teaching materials, this curriculum is portable and can likely be used at other institutions without significant barriers.

Limitations of our curriculum include that it was limited to one session. While this can make it easier to implement, skills and knowledge will improve and are more likely to be sustained with further educational interventions. Additionally, we only evaluated at short-term outcomes using a posttest immediately following the session. This curriculum could be further evaluated by assessing knowledge retention at more distant intervals from the initial training. Our evaluation instrument was not piloted prior to implementation, and we have not assessed for changes in clinical practice based on the education provided. Further evaluation of this same curriculum could occur by measuring a change in practice patterns for EM interns and for our fourth-year medical students once they enter residency.

Lessons learned during the implementation of this workshop include the need for further discussion and dissemination of supplemental materials and adjusting the number of facilitators based on the learners' level of training. As this is such a new topic to EM, we found many of the learners were completely unfamiliar with the COWS[13] and the validated screening tools. We found that when paper copies of these materials were supplied, the learners seemed to have better retention and could then use these materials when working though the case. Additionally, we found that the additional facilitators were helpful when delivering the workshop to the EM interest group, which included first- and second-year medical students. However, in all other sessions that were exclusively for EM-bound fourth-year students and EM interns, one facilitator was adequate to assist learners through the case. Similarly, we found the workshop was better suited for EM-bound fourth-year students and EM interns, as compared to the cohort from the EM interest group meeting. We found it challenging to deliver this workshop to first- and second-year medical students, who did not seem to have the clinical experience to gain all the educational points the workshop offered.

In summary, we describe the successful use of a 1-hour curriculum to introduce EM-bound fourth-year medical students and EM interns to ED-based interventions for OUD. As we continue to educate our students, residents, and fellow faculty about new, innovative, and evidenced-based treatment strategies for OUD, we can continue to improve our quality of care delivered to patients and better address the opioid epidemic.

Appendices

A. OUD in the ED Introduction.pptx

B. OUD Case - Facilitator.docx

C. OUD Case - Trainee.docx

D. Test Questions.docx

E. Test Questions Answer Key.docx

All appendices are peer reviewed as integral parts of the Original Publication.

Lindsey Jennings, MD, MPH: Assistant Professor, Department of Emergency Medicine, Medical University of South Carolina

Tessa Warner, MD, MPH: Resident Physician, Department of Emergency Medicine, Medical University of South Carolina

Bastien Bacro-Duverger, MD: Resident Physician, Department of Emergency Medicine, University of Alabama at Birmingham

Copyright © 2020 Jennings et al. This is an open-access publication distributed under the terms of the Creative Commons Attribution license.

Disclosures
None to report.

Funding/Support
None to report.

Ethical Approval
Reported as not applicable.

References

1. Center for Behavioral Health Statistics and Quality. Key substance use and mental health indicators in the United States: results from the 2015 National Survey on Drug Use and Health. Substance Abuse and Mental Health Services Administration website. https://www.samhsa.gov/data/sites/default/files/NSDUH-FFR1-2015Rev1/NSDUH-FFR1-2015Rev1/NSDUH-FFR1-2015Rev1/NSDUH-National%20Findings-REVISED-2015.pdf. Published September 2016. Accessed March 3, 2019.
2. Lipari RN, Williams MR, Copello EAP, et al. Risk and protective factors and estimates of substance use initiation: results from the 2015 National Survey on Drug Use and Health. Substance Abuse and Mental Health Services Administration website. https://www.samhsa.gov/data/sites/default/files/NSDUH-PreventionandInit-2015/NSDUH-PreventionandInit-2015.pdf. Published October 2016. Accessed March 3, 2019.
3. Hedegaard H, Warner M, Miniño AM. *Drug Overdose Deaths in the United States, 1999-2016*. Hyattsville, MD: US Department of Health and Human Services, Centers for Disease Control, National Center for Health Statistics; 2017. NCHS Data Brief No. 294.
4. Rudd RA, Aleshire N, Zibell JE, Gladden RM. Increases in drug and opioid overdose deaths—United States, 2000-2014. *MMWR Morb Mortal Wkly Rep*. 2016;64(50-51):1378-1382. https://doi.org/10.15585/mmwr.mm6450a3
5. HCUP Fast Stats—opioid-related hospital use. Healthcare Cost and Utilization Project website. https://www.hcup-us.ahrq.gov/faststats/OpioidUseServlet. Updated October 30, 2018.
6. Weiner SG, Baker O, Bernson D, Schuur JD. 402: One-year mortality of opioid overdose victims who receive naloxone by emergency medical services [abstract]. *Ann Emerg Med*. 2017;70(4)(suppl):S158. https://doi.org/10.1016/j.annemergmed.2017.07.281
7. Jones CM, Campopiano M, Baldwin G, McCance-Katz E. National and state treatment need and capacity for opioid agonist medication-assisted treatment. *Am J Public Health*. 2015;105(8):e55-e63. https://doi.org/10.2105/AJPH.2015.302664
8. D'Onofrio G, O'Connor PG, Pantalon MV, et al. Emergency department-initiated buprenorphine/naloxone treatment for opioid dependence: a randomized clinical trial. *JAMA*. 2015;313(16):1636-1644. https://doi.org/10.1001/jama.2015.3474
9. Keenan MP, Schenker KA, Sarsfield MJ. A complicated opioid overdose: a simulation for emergency medicine residents. *MedEdPORTAL*. 2017;13:10616. https://doi.org/10.15766/mep_2374-8265.10616
10. Soskin P, Duong D. Social worker as SBIRT instruction to emergency medicine residents. *MedEdPORTAL*. 2014;10:9840. https://doi.org/10.15766/mep_2374-8265.9840
11. Butler SF, Fernandez K, Benoit C, Budman SH, Jamison RN. Validation of the revised Screener and Opioid Assessment for Patients With Pain (SOAPP-R). *J Pain*. 2008;9(4):360-372. https://doi.org/10.1016/j.jpain.2007.11.014
12. Resource Guide: Screening for Drug Use in General Medical Settings—the NIDA Quick Screen. National Institute on Drug Abuse website. https://www.drugabuse.gov/publications/resource-guide-screening-drug-use-in-general-medical-settings/nida-quick-screen. Updated March 2012. Accessed September 2019.
13. Wesson DR, Ling W. The Clinical Opiate Withdrawal Scale (COWS). *J Psychoactive Drugs*. 2011;35(2):253-259. https://doi.org/10.1080/02791072.2003.10400007

Received: September 27, 2019
Accepted: October 14, 2019
Published: May 15, 2020

Copyright © 2020 Jennings et al. This is an open-access publication distributed under the terms of the Creative Commons Attribution license.
